# Supplementary material for: Synchrony, oscillations, and phase relationships in collective neuronal activity: A highly comparative overview of methods
Source: PLoS Comput Biol. 2025 Oct 24;21(10):e1013597. doi: 10.1371/journal.pcbi.1013597 (PMC12574956; doi:10.1371/journal.pcbi.1013597)
Supplement: S1 Text — (PDF) [file pcbi.1013597.s001.pdf]

Supporting Information for

Synchrony, oscillations, and phase relationships in collective neuronal activity: a highly comparative overview of methods

Fabiano Baroni, Ben D. Fulcher

Supplementary Methods

Multineuron spike train measures: supplementary details

All MSTMs have been implemented as described in the original publication, using the code provided by the authors when available, with the exception of the Tiesinga–Sejnowski synchrony measure $\mathrm{S_{TS}}$ [1] which has been implemented as

$$\mathrm{S_{TS}} = \frac{1}{\sqrt{N}} \left(\frac{\sqrt{\langle \tau_\nu^2 \rangle - \langle \tau_\nu \rangle^2}}{\langle \tau_\nu \rangle} \right) , \tag{1}$$

where N is the number of neurons, and τ_ν are obtained from the combined set of time-ordered spike times t_ν as $\tau_\nu = t_{\nu+1} - t_\nu$. We use this formulation instead of

$$\mathrm{S_{TS}} = \frac{1}{\sqrt{N}} \left(\frac{\sqrt{\langle \tau_\nu^2 \rangle - \langle \tau_\nu \rangle^2}}{\langle \tau_\nu \rangle} - 1 \right) , \tag{2}$$

as reported in the original publication, in order to yield values that are always ≥ 0 and are included in [0,1] in most cases.

In the case of Golomb–Rinzel synchrony $\mathrm{S_{GR}}$ [2], this measure was originally intended to quantify synchrony at the level of continuous variables (voltage trajectories). We adapted it to the analysis of point processes by first convolving the spike time series with a Gaussian kernel, and then applying the original measure to the resulting continuous traces.

The measures $\mathrm{S_{QQ}}$ and $\mathrm{S_{qq}}$ [3] were modified by counting at most one spike event t_j^y in spike train y occurring shortly after a reference spike event t_i^x in spike train x. This ensures that the measures are normalized in the intervals [0,1] and [−1,1], respectively, for any value of the synchrony timescale τ. We note that the authors proposed a method for estimating a suitable value for the synchrony timescale from the data: setting it equal to half the local minimum inter-spike interval. This choice prevents double-counting and hence ensures that the measures are included in the intervals indicated above. However, here we prefer to vary the synchrony timescale in an interval in analogy with other measures and also considering that evaluation of these measures for a range of timescales is a common approach for estimating the relevant timescales from the data [4,5]. We also note that it is possible to set τ based on the same criterion (or a corresponding global criterion) for any measure that includes a timescale parameter.

Some of the measures derive from the power spectrum of the instantaneous population firing rate, obtained by convolving each spike train with a Gaussian kernel of bandwidth 0.1 ms, averaging across neurons, and z-scoring across time. The power spectrum was estimated using a multi-taper method [6] implemented in the Chronux data analysis toolbox with a time-bandwidth product of 10 and 19 tapers, resulting in a relatively smooth power estimation that is suitable for spectral feature extraction and

parameterization. In particular, we extracted the amplitude and frequency of the log power spectrum maximum. Extraction of these and other features from the power spectrum of the local field potential or other aggregate signals is a common method of feature extraction in the neurosciences (see, for example, [7]), and similar approaches extract features from the auto- or cross-correlograms [8–11]. We also performed a FOOOF (fitting oscillations & one over f) parameterization of the power spectrum of the instantaneous population firing rate [12], whereby the log power spectral density was described by the sum of an aperiodic component and one or more periodic components:

$$\mathrm{PSD}(f) = L(f) + \sum_{n=0}^{N_p} G_n \,, \tag{3}$$

where $L(f)$ represents the aperiodic component, described as a power-law function with an offset

$$L(f) = b_L - \log f^{\chi_L} \,, \tag{4}$$

and each G_n is a Gaussian fit to a peak:

$$\mathcal{G}(f) = A_{\mathcal{G}} \mathrm{e}^{-\frac{1}{2}\left(\frac{f - f_{\mathcal{G}}}{\sigma_{\mathcal{G}}}\right)^2} . \tag{5}$$

This fit resulted in an estimation of the parameters in the expressions above (b_L and χ_L for the aperiodic component, and $A_{\mathcal{G}}$, $f_{\mathcal{G}}$ and $\sigma_{\mathcal{G}}$ for each peak), as well as in parameters that measure the goodness of fit (the mean absolute error MAE and the coefficient of determination R^2). Another measure was obtained for each peak by combining the Gaussian parameters as follows:

$$\beta_{\mathcal{G}} = \frac{A_{\mathcal{G}} f_{\mathcal{G}}}{\sigma_{\mathcal{G}}} . \tag{6}$$

We performed FOOOF parameterization with the number of peaks N_p set to 1 and 2. An additional feature representing the fit improvement when considering 2 peaks versus one was defined as $R^2_{\mathrm{ratio}} = R^2_{N_p=2}/R^2_{N_p=1}$. The threshold for a peak to be included in the model was set to 3 standard deviations (SD) of the residuals after subtraction of the aperiodic component. This threshold is more conservative than the default threshold of 2 SD included in the original FOOOF code, but resulted in the detection of at least one spectral peak in a similar number of cases when applied to synthetic spike trains. Peaks detected only at the 2 SD threshold often appeared spurious upon visual inspection, suggesting that the 3 SD threshold provides a better balance between identifying meaningful peaks and excluding false positives. Below this threshold, the corresponding parameters were excluded from subsequent processing. These amount to a maximum of 4 (12) MSTMs in the core (extended) set of measures. The number and proportion of spike trains with at least one excluded MSTM for each synthetic spike train family and biological datasets are reported in Table 1. Gaussian fitting of the power spectrum (with or without an aperiodic term describing the $1/f$ component) is also a common technique of feature extraction in the neurosciences, particularly in the EEG literature (e.g., [13–15]).

Biological spike train analysis

To ensure that the results obtained using synthetic spike trains do not depend critically on idiosyncratic aspects of the spike train generation algorithms, and to illustrate the benefits of a highly comparative analysis for the visualization, organization and classification of real spike trains, we applied the same battery of MSTMs to biological spike train datasets. For each dataset, we applied the same preprocessing scheme, which is detailed below. Information on each dataset is briefly reported below and summarized in Table 2.

Table 1. Number of spike trains for each synthetic spike train family and biological dataset.

name	$N_{\text{win}}^{\text{tot}}$	$N_{\text{win}}^{\text{rej}}$, core set	$N_{\text{win}}^{\text{rej}}$, extended set	$N_{\text{win}}^{\text{rej}}/N_{\text{win}}^{\text{tot}}$, core set	$N_{\text{win}}^{\text{rej}}/N_{\text{win}}^{\text{tot}}$, extended set
**Synthetic spike train families**					
single-scale	450	21	52	4.67%	11.56%
dual-scale	450	124	190	27.56%	42.22%
**Biological datasets**					
rat A1	280	6	8	2.14%	2.86%
mouse CA1	800	219	296	27.37%	37%
monkey V1	294	255	257	86.73%	87.41%

$N_{\text{win}}^{\text{tot}}$, total number of time windows; $N_{\text{win}}^{\text{rej}}$, number of time windows with at least one excluded MSTM.

Table 2. Biological spike train datasets considered in this study.

dataset	species	brain area	brain state	stimulation	# of animals	# of recordings	$N_{\text{neu}}^{\text{min}}$	recording duration	N_{win}	$N_{\text{win}}^{\text{tot}}$
See et al. (2018a) [16]; See et al. (2018b) [17]	rat (Sprague–Dawley)	A1	ketamine/ xylazine anesthesia	dynamic broadband auditory stimulation	not reported	14/16	27	10 min	20	280
Formozov et al. (2022a) [18]; Formozov et al. (2022b) [19]	mouse (C57BL/6J)	dorsal CA1	wakefulness & natural sleep	spontaneous activity	3	4	28	1–2 h	134–244	800
Smith and Kohn (2008) [20]; Kohn and Smith (2016) [21]	monkey (*Macaca fascicularis*)	V1	sufentanil anesthesia	uniform gray screen	4	6	54	15–31 min	30–62	294

$N_{\text{neu}}^{\text{min}}$, minimum number of neurons across recordings; N_{win}, number of time windows per recording; $N_{\text{win}}^{\text{tot}}$, total number of time windows.

Biological spike train preprocessing

Putative single-units with a high proportion of very short ISIs (i.e., with 5% or more of ISIs < 1 ms) or with long periods of silence ($\max(\text{ISI}) > 20$ s) were discarded. Then, spikes that resulted in one or more consecutive ISI < 1 ms were removed as follows, depending on the number n_{shortISI} of consecutive short ISIs: *i*) $n_{\text{shortISI}} = 1$: the second spike of the doublet was removed; *ii*) $n_{\text{shortISI}} = 2$: the first and third spike of the triplet were removed; and *iii*) $n_{\text{shortISI}} = 3$: the first, third and fourth spike of the quadruplet were removed. There were no cases with $n_{\text{shortISI}} > 3$.

Then, for each dataset, we homogenized the number of neurons across recordings by subsampling neurons from those recordings with a number of neurons higher than the minimum number of neurons across recordings. That is, if $N_{\text{neu}}^{\text{min}}$ is the minimum number of neurons across recordings in a dataset, we

randomly subsampled $N_{\text{neu}}^{\text{min}}$ neurons without replacement for each recording to obtain a dataset that is homogeneous with respect to the number of simultaneously acquired single-units. The battery of MSTMs was applied to each 30 s non-overlapping window, yielding N_{win} time windows per recording. In the case of the rat auditory cortex dataset, 2 of the 16 recordings had a number of neurons considerably lower than the others and were discarded.

Rat auditory cortex. We analyzed dense single-unit recordings from the primary auditory cortex (A1) of female Sprague–Dawley rats anesthetized with ketamine/xylazine during the presentation of dynamic broadband stimuli [16], obtained from [17]. Briefly, recordings were made using a 32- or 64-channel probe, inserted perpendicular to the cortical surface to a depth of approximately 800 or 1400 µm respectively using a microdrive. Neural traces were band-pass filtered between 500 and 6000 Hz and were recorded to disk at 20 kHz sampling rate, and then spike-sorted offline. The total duration for each recording was 600 s. See [17] for more details on this dataset.

Mouse dorsal hippocampus. We analyzed electrophysiological recordings acquired with a silicon probe inserted in the dorsal CA1 to a depth of 1.6 mm in three C57BL/6J mice of both sexes [18], obtained from [19]. The signal was acquired with an extracellular amplifier at 32 kHz sampling rate and with a band-pass filter in the 0.1–8000 Hz range.

Monkey visual cortex. We analyzed single-unit activity recorded using Utah arrays placed in the primary visual cortex (V1) of four adult male *Macaca fascicularis* monkeys, anesthetized with sufentanil, using a total of six arrays [20], obtained from [21]. The array was inserted to a depth of 0.6 mm into the cortex, resulting in recordings confined mostly to layers 2–3.

Multivariate classification analyses

To characterize the informational features borne by each MSTM, we applied a classification analysis to decode brain state in one of the datasets [18], which also included sleep scoring data for each 10 s time window with 50% overlap. Each time window was classified as wake, REM or NREM, or left unclassified, based on electrophysiological data not used here (LFP and EMG) and movement data (see [22] for details). To yield sleep scoring data with the same temporal resolution as the MSTMs, we combined adjacent 10 s time windows in groups of 5, each group corresponding to a 30 s spike train window. After each group of 5 windows, the next 10 s sleep scoring window was skipped, before the next group of 5 windows was considered. Each 30 s window was classified in one of the 3 categories if at least 3 out of the 5 comprising 10 s windows were classified in that category, and none was classified in any other category. This procedure yielded few REM windows; hence, we focused our classification analysis on discriminating between wake vs. NREM spike trains. The number of resulting time windows in each class for each recording is shown in Table 3.

We estimated the amount of information conveyed by neural signals using binary Regularized Least-Square Classifiers (RLSC, [23]) with regularization parameter $\lambda = 10^6$. Regularized Least-Square Classification is a machine learning technique that estimates the linear separability between patterns according to their class.

We performed 2 different types of decoding analysis: *i*) univariate decoding, where each MSTM was considered individually, and *ii*) bivariate decoding, where each pair of MSTMs was considered as input patterns to the classifiers. Each type of decoding analysis was performed both within each recording as well as across recordings, in a leave-one-recording-out (LORO) cross-validation scheme.

A set of weights that optimally separate samples (i.e., time windows) according to their class was determined using a subset of the available samples, denoted as training set. The performance of the classifier was defined using a different set of samples, denoted as test set, as the area under the Receiver

Table 3. Number of time windows in each class for each recording.

recording	$N_{\mathrm{win}}^{\mathrm{wake}}$	$N_{\mathrm{win}}^{\mathrm{NREM}}$	$N_{\mathrm{win}}^{\mathrm{wake,non-rej}}$	$N_{\mathrm{win}}^{\mathrm{NREM,non-rej}}$
M1R1	58	55	19	29
M1R2	61	28	33	23
M2R2	22	13	12	11
M4R1	33	71	15	33

Number of time windows in each class for each recording of the mouse dataset [19] used for the brain state decoding analysis in section "Brain state classification from spike train measures". $N_{\mathrm{win}}^{\mathrm{wake,non-rej}}$ and $N_{\mathrm{win}}^{\mathrm{NREM,non-rej}}$ indicate the number of time windows classified as wake and NREM, respectively, where no MSTM was rejected.

Operating Characteristic (ROC) curve, which we refer to as A$'$ (A prime). We report the average A$'$ values over N_{iter} cross-validations. For within-recording decoding, in each cross-validation, we randomly chose a set of $0.7 \times \min(N_1, N_2)$ (rounded to the nearest integer) samples of each class as the training set, where N_1 and N_2 are the number of samples in class 1 and 2, respectively. As the test set, we chose $\min(N_1, N_2) - \mathrm{round}(0.7 \times \min(N_1, N_2))$ samples of each class among those that were not already included in the training set. Before being fed to the classifier, inputs were z-transformed: the mean and standard deviation of each MSTM in the training set was calculated, and used to transform both training and test sets. Then, optimal RLSC weights were estimated using training samples, and their capacity to separate test samples according to their class was measured as the area under the ROC curve (A$'$).

For LORO decoding, in each outer iteration, one recording was set as the test recording, and the other recordings as training recordings. Then, the average A$'$ values over N_{iter} cross-validations was calculated similarly as described above, but with the difference that, in each cross-validation, N_{min} randomly chosen samples of each training recording and each class were included in the training set, and N_{min} randomly chosen samples of the test recording and each class were included in the test set, where N_{min} is the minimum number of samples across recordings and classes. In addition to the average A$'$ value over cross-validations for each test recording, we also report their median values across test recordings. The number of cross-validations N_{iter} was set to 1000 for all the decoding analyses.

For each pair of MSTMs (X,Y), decoding synergy $\mathcal{S}_{\mathrm{A}'_{\mathrm{X,Y}}}$ was defined as the percentage change in decoding accuracy obtained when considering both MSTMs jointly, compared to the maximal decoding accuracy obtained when considering each MSTM individually: $\mathcal{S}_{\mathrm{A}'_{\mathrm{X,Y}}} = (\mathrm{A}'_{\mathrm{X,Y}} - \max(\mathrm{A}'_{\mathrm{X}}, \mathrm{A}'_{\mathrm{Y}}))/\max(\mathrm{A}'_{\mathrm{X}}, \mathrm{A}'_{\mathrm{Y}})$.

Significance of A$'$ values was estimated via permutation-based statistics. For each classification considered, the class labels were randomly shuffled. In the case of LORO decoding, shuffling was performed within each recording, to destroy any information related to class labels but preserve any recording-specific idiosyncrasy. Then, the average A$'$ value over N_{iter} realizations of training and test sets was calculated as described above. This procedure was repeated N_{perm} times, where $N_{\mathrm{perm}} = 1000$ (100) for univariate (bivariate) decoding, yielding a probability distribution of average A$'$ values corresponding to the null hypothesis of lack of separability between the two classes. An empirical average A$'$ value was considered significant at level p if it exceeded the p-percentile of the corresponding null distribution. For univariate decoding, significance thresholds at $p = 0.01$ were estimated separately for each classification considered. In order to improve the estimation of higher significance thresholds and in the case of bivariate decoding, null A$'$ values were pooled across MSTMs or MSTM pairs, and the corresponding significance thresholds were calculated from the resulting null distribution.

Analyses of the relationships between measures and between time windows

Hierarchical clustering of measures

To characterize the structure of the space of MSTMs in terms of their empirical similarity over a broad set of spike trains, and to detect any cluster structure in that space at multiple scales, we conducted hierarchical agglomerative clustering analyses, similarly to previous studies that adopted the highly comparative approach [24, 25]. Each MSTM was represented by a vector of values resulting from its application to each spike train considered. As a distance metric, we used $D = 1 - |\rho|$, where ρ is the Spearman correlation value between each pair of MSTMs. The choice to use this distance metric, based on the absolute value $|\rho|$, was motivated by the fact that the majority of synchrony measures increase with synchrony, while some of them decrease. With this distance metric, MSTMs that covary across the dataset will be considered as similar regardless of the sign of the variation.

Then, an iterative procedure was conducted to group MSTMs into a hierarchical cluster tree or dendrogram, using MATLAB's `linkage` function. We started with each MSTM as its own cluster. Then, the two closest clusters were joined together into a single cluster, and this procedure repeated iteratively until all the MSTMs were grouped into one cluster. The distance between two clusters A and B was defined as the average among the distances between all pairs of MSTMs (S_i, S_j), where $S_i \subseteq A$ and $S_j \subseteq B$ (average linkage clustering).

Hierarchical clustering of time windows

Biological spiking data exhibit variations in multiple features of individual and collective coordination unfolding over multiple timescales. To organize and visualize the structure of neuronal activity and coordination patterns in biological spiking data, as captured by our MSTM library, we conducted hierarchical agglomerative clustering analyses on time windows, similarly as performed on MSTMs. Each time window was described by a feature vector resulting from the application of each MSTM. As a distance metric, we used $D = 1 - \rho$, where ρ is the Spearman correlation value between each pair of time windows. Linkage was performed using the average linkage criterion.

Recording fingerprinting

To quantify recording-wise distinctiveness, that is, the extent to which time windows from the same recording tend to be more similar to each other than to windows from different recordings, we calculated the silhouette score $\mathcal{S}$ [26]. The silhouette score $\mathcal{S}_i$ is defined for each data point (here, for each spike train corresponding to a specific time window, as described by a feature vector resulting from the application of each MSTM) as

$$\mathcal{S}_i = \frac{b_i - a_i}{\max(a_i, b_i)}\,, \tag{7}$$

where a_i is the mean distance between the data point and all other points in the same class (here, from the same recording), and b_i is the distance between that point and its nearest neighbor from a different class. The silhouette score ranges from -1 to 1, and approaches 1 if $a_i \ll b_i$. We averaged the silhouette scores across time windows to obtain a measure $\mathcal{S}$ of the average level of recording-wise distinctiveness for each dataset: $\mathcal{S} = \frac{1}{n}\sum_1^n \mathcal{S}_i$. We calculated the silhouette score for both the core and the extended sets of MSTMs, as well as for their 2D MDS representation, and adopted the Euclidean distance (after z-scoring across windows) as distance metric.

To assess if empirical $\mathcal{S}$ values are different from what would be expected by chance, we constructed a null distribution of $\mathcal{S}$ values by randomly shuffling recording labels and computing the resulting $\mathcal{S}$. This procedure was repeated $N_{\text{perm}} = 1000$ times, yielding a probability distribution of S values corresponding to the null hypothesis of lack of recording-wise distinctiveness, that is, recording-wise homogeneity. An

empirical S value was considered significant at $p = 0.001$ if it exceeded the p-percentile of the corresponding null distribution.

Dimensionality reduction

In order to visualize the patterns of similarity between different time windows in a two-dimensional space, as well as to assess how recording-wise distinctiveness is affected by a two-dimensional projection of MSTMs, we performed multidimensional scaling (MDS) using $D = 1 - \rho$ as a measure of dissimilarity between pairs of vectors. MDS enables one to represent the original, high-dimensional data (corresponding to one dimension for each MSTM) in a lower dimensional space while approximately preserving the relative distances between data points [27].

Dimensionality estimation

Many measures included in our MSTM library are meant to capture the same or similar phenomena, hence we expect subsets of MSTMs to display strong similarities. Correspondingly, the high-dimensional representation provided by the MSTM library is expected to lie on a lower dimensional submanifold. To assess the dimensionality of such lower dimensional submanifold, we estimated the intrinsic dimensionality (ID) of the highly comparative data matrix (where each row corresponds to a MSTM and each column to a spike train) using both linear and nonlinear approaches [28, 29]. The linear approach is based on Principal Component Analysis (PCA) decomposition: PCA identifies mutually orthogonal directions in the space of MSTMs and ranks them in decreasing order of associated variance. The dimensionality is then defined as the minimum number of PCA components required to reach a predetermined threshold of cumulative variance. We considered two different thresholds: 95% and 99% of the total variance.

The nonlinear approaches are geometrical methods based on nearest neighbor (NN) statistics [30, 31], implemented in [32]. These methods are meant to be applied in a range of scales: at each scale, determined by the neighbors' rank and/or by the number of data points that enter the calculation, they return the estimated number of "soft" directions, that is, the number of directions in which the features of the dataset change remarkably, as opposed to "noise" directions characterized by small variations. Nearest neighbor based intrinsic dimension methods have been shown to yield accurate dimensionality estimates with fewer samples than required by comparable methods [31, 33].

Supplementary Discussion

Directions for future developments

This study focuses on collective patterns of multineuron coordination such as synchrony, oscillations, and phase relationships. As such, we did not consider measures of spike train burstiness. Nonetheless, bursting activity is well known to be an important component of the neural code [34, 35], and several methods have been proposed for the quantification of burstiness as well as for the generation of synthetic spike trains that comprise burst spiking [36–41]. The inclusion of these methods in future libraries is expected to yield an even richer description of collective firing patterns and enhance our understanding of the relationships between bursting measures and the MSTMs considered here, particularly with respect to the univariate measures of firing variability.

While we limited our investigation to spike train measures that return a single scalar value, future developments could also consider methods that return multiple values, including algorithms to characterize firing patterns [42–44]; to estimate connectivity or causal relationships from multineuron spike trains (e.g., [45–47]), including high-order interdependencies [48]; to detect neuronal assemblies (e.g., [49–53]) or non-random spike sequences embedded in them (e.g., [54–59]); and to estimate their geometrical properties (e.g., [60, 61]). Suitable modifications of the highly comparative methodology employed here will be useful

to characterize similarities and differences among these methods and their variants, and their relationships with the scalar MSTMs employed in this work.

References

1. Tiesinga PH, Sejnowski TJ. Rapid Temporal Modulation of Synchrony by Competition in Cortical Interneuron Networks. Neural Computation. 2004;16(2):251–275. doi:10.1162/089976604322742029.

2. Golomb D, Rinzel J. Dynamics of Globally Coupled Inhibitory Neurons with Heterogeneity. Physical Review E. 1993;48(6):4810–4814. doi:10.1103/PhysRevE.48.4810.

3. Quian Quiroga R, Kreuz T, Grassberger P. Event Synchronization: A Simple and Fast Method to Measure Synchronicity and Time Delay Patterns. Physical Review E. 2002;66(4):041904. doi:10.1103/PhysRevE.66.041904.

4. Machens CK, Schütze H, Franz A, Kolesnikova O, Stemmler MB, Ronacher B, et al. Single Auditory Neurons Rapidly Discriminate Conspecific Communication Signals. Nature Neuroscience. 2003;6(4):341–342. doi:10.1038/nn1036.

5. Narayan R, Graña G, Sen K. Distinct Time Scales in Cortical Discrimination of Natural Sounds in Songbirds. Journal of Neurophysiology. 2006;96(1):252–258. doi:10.1152/jn.01257.2005.

6. Mitra P, Bokil H. Observed Brain Dynamics. 1st ed. Oxford University Press, USA; 2007. doi:10.1093/acprof:oso/9780195178081.001.0001.

7. Miller WL, Sigvardt KA. Spectral Analysis of Oscillatory Neural Circuits. Journal of Neuroscience Methods. 1998;80(2):113–128. doi:10.1016/S0165-0270(97)00185-4.

8. König P. A Method for the Quantification of Synchrony and Oscillatory Properties of Neuronal Activity. Journal of Neuroscience Methods. 1994;54(1):31–37. doi:10.1016/0165-0270(94)90157-0.

9. Schneider G, Havenith MN, Nikolić D. Spatiotemporal Structure in Large Neuronal Networks Detected from Cross-Correlation. Neural Computation. 2006;18(10):2387–2413. doi:10.1162/neco.2006.18.10.2387.

10. Mureşan RC, Jurjuţ OF, Moca VV, Singer W, Nikolić D. The Oscillation Score: An Efficient Method for Estimating Oscillation Strength in Neuronal Activity. Journal of Neurophysiology. 2008;99(3):1333–1353. doi:10.1152/jn.00772.2007.

11. Nikolić D, Mureşan RC, Feng W, Singer W. Scaled Correlation Analysis: A Better Way to Compute a Cross-Correlogram. European Journal of Neuroscience. 2012;35(5):742–762. doi:10.1111/j.1460-9568.2011.07987.x.

12. Donoghue T, Haller M, Peterson EJ, Varma P, Sebastian P, Gao R, et al. Parameterizing Neural Power Spectra into Periodic and Aperiodic Components. Nature Neuroscience. 2020;23(12):1655–1665. doi:10.1038/s41593-020-00744-x.

13. Nakamura M, Nishida S, Neshige R, Shibasaki H. Quantitative Analysis of 'Organization' by Feature Extraction of the EEG Power Spectrum. Electroencephalography and Clinical Neurophysiology. 1985;60(1):84–89. doi:10.1016/0013-4694(85)90954-X.

14. Neto E, Allen EA, Aurlien H, Nordby H, Eichele T. EEG Spectral Features Discriminate between Alzheimer's and Vascular Dementia. Frontiers in Neurology. 2015;6:25. doi:10.3389/fneur.2015.00025.

15. Wen H, Liu Z. Separating Fractal and Oscillatory Components in the Power Spectrum of Neurophysiological Signal. Brain Topography. 2016;29(1):13–26. doi:10.1007/s10548-015-0448-0.

16. See JZ, Atencio CA, Sohal VS, Schreiner CE. Coordinated Neuronal Ensembles in Primary Auditory Cortical Columns. eLife. 2018;7. doi:10.7554/eLife.35587.

17. See JZ, Atencio CA, Schreiner CE. High-Density Extracellular Recordings from the Primary Auditory Cortex in Anesthetized Rats Listening to Dynamic Broadband Stimuli. CRCNS.org. 2018. doi:10.6080/K09021X1.

18. Formozov A, Chini M, Dieter A, Yang W, Pöpplau JA, Hanganu-Opatz IL, et al. Calcium Imaging and Electrophysiology of Hippocampal Activity under Anesthesia and Natural Sleep in Mice. Scientific Data. 2022;9(1):113. doi:10.1038/s41597-022-01244-2.

19. Formozov A, Chini M, Dieter A, Yang W, Pöpplau JA, Hanganu-Opatz IL, et al. Calcium Imaging and Electrophysiology of Hippocampal Activity under Anesthesia and Natural Sleep in Mice. G-Node. 2022. doi:10.12751/g-node.lkx6kk.

20. Smith MA, Kohn A. Spatial and Temporal Scales of Neuronal Correlation in Primary Visual Cortex. The Journal of Neuroscience. 2008;28(48):12591–12603. doi:10.1523/jneurosci.2929-08.2008.

21. Kohn A, Smith MA. Utah Array Extracellular Recordings of Spontaneous and Visually Evoked Activity from Anesthetized Macaque Primary Visual Cortex (V1). CRCNS.org. 2016. doi:10.6080/K0NC5Z4X.

22. Yang W, Chini M, Pöpplau JA, Formozov A, Dieter A, Piechocinski P, et al. Anesthetics Fragment Hippocampal Network Activity, Alter Spine Dynamics, and Affect Memory Consolidation. PLOS Biology. 2021;19(4):e3001146. doi:10.1371/journal.pbio.3001146.

23. Rifkin R, Yeo G, Poggio T. Regularized Least-Squares Classification. Nato Science Series Sub Series III Computer and Systems Sciences. 2003;190:131–154.

24. Fulcher BD, Little MA, Jones NS. Highly Comparative Time-Series Analysis: The Empirical Structure of Time Series and Their Methods. Journal of The Royal Society Interface. 2013;10(83):20130048. doi:10.1098/rsif.2013.0048.

25. Cliff OM, Bryant AG, Lizier JT, Tsuchiya N, Fulcher BD. Unifying Pairwise Interactions in Complex Dynamics. Nature Computational Science. 2023;3(10):883–893. doi:10.1038/s43588-023-00519-x.

26. Rousseeuw PJ. Silhouettes: A Graphical Aid to the Interpretation and Validation of Cluster Analysis. Journal of Computational and Applied Mathematics. 1987;20:53–65. doi:10.1016/0377-0427(87)90125-7.

27. Cox TF, Cox MAA. Multidimensional Scaling, Second Edition. CRC Press; 2000. doi:10.1201/9780367801700.

28. Campadelli P, Casiraghi E, Ceruti C, Rozza A. Intrinsic Dimension Estimation: Relevant Techniques and a Benchmark Framework. Mathematical Problems in Engineering. 2015;2015:e759567. doi:10.1155/2015/759567.

29. Camastra F, Staiano A. Intrinsic Dimension Estimation: Advances and Open Problems. Information Sciences. 2016;328:26–41. doi:10.1016/j.ins.2015.08.029.

30. Facco E, d'Errico M, Rodriguez A, Laio A. Estimating the Intrinsic Dimension of Datasets by a Minimal Neighborhood Information. Scientific Reports. 2017;7(1):12140. doi:10.1038/s41598-017-11873-y.

31. Denti F, Doimo D, Laio A, Mira A. The Generalized Ratios Intrinsic Dimension Estimator. Scientific Reports. 2022;12(1):20005. doi:10.1038/s41598-022-20991-1.

32. Glielmo A, Macocco I, Doimo D, Carli M, Zeni C, Wild R, et al. DADApy: Distance-based Analysis of Data-Manifolds in Python. Patterns. 2022;3(10):100589. doi:10.1016/j.patter.2022.100589.

33. Altan E, Solla SA, Miller LE, Perreault EJ. Estimating the Dimensionality of the Manifold Underlying Multi-Electrode Neural Recordings. PLOS Computational Biology. 2021;17(11):e1008591. doi:10.1371/journal.pcbi.1008591.

34. Zeldenrust F, Wadman WJ, Englitz B. Neural Coding with Bursts—Current State and Future Perspectives. Frontiers in Computational Neuroscience. 2018;12:48. doi:10.3389/fncom.2018.00048.

35. Friedenberger Z, Harkin E, Tóth K, Naud R. Silences, Spikes and Bursts: Three-Part Knot of the Neural Code. The Journal of Physiology. 2023;601(23):5165–5193. doi:10.1113/JP281510.

36. Gourévitch B, Eggermont JJ. A Nonparametric Approach for Detection of Bursts in Spike Trains. Journal of Neuroscience Methods. 2007;160(2):349–358. doi:10.1016/j.jneumeth.2006.09.024.

37. Bingmer M, Schiemann J, Roeper J, Schneider G. Measuring Burstiness and Regularity in Oscillatory Spike Trains. Journal of Neuroscience Methods. 2011;201(2):426–437. doi:10.1016/j.jneumeth.2011.08.013.

38. Ko D, Wilson CJ, Lobb CJ, Paladini CA. Detection of Bursts and Pauses in Spike Trains. Journal of Neuroscience Methods. 2012;211(1):145–158. doi:10.1016/j.jneumeth.2012.08.013.

39. Carroll MS, Ramirez JM. Cycle-by-Cycle Assembly of Respiratory Network Activity Is Dynamic and Stochastic. Journal of Neurophysiology. 2013;109(2):296–305. doi:10.1152/jn.00830.2011.

40. Eisenman LN, Emnett CM, Mohan J, Zorumski CF, Mennerick S. Quantification of Bursting and Synchrony in Cultured Hippocampal Neurons. Journal of Neurophysiology. 2015;114(2):1059–1071. doi:10.1152/jn.00079.2015.

41. Cotterill E, Charlesworth P, Thomas CW, Paulsen O, Eglen SJ. A Comparison of Computational Methods for Detecting Bursts in Neuronal Spike Trains and Their Application to Human Stem Cell-Derived Neuronal Networks. Journal of Neurophysiology. 2016;116(2):306–321. doi:10.1152/jn.00093.2016.

42. Fellous JM, Tiesinga PHE, Thomas PJ, Sejnowski TJ. Discovering Spike Patterns in Neuronal Responses. Journal of Neuroscience. 2004;24(12):2989–3001. doi:10.1523/JNEUROSCI.4649-03.2004.

43. Kumbhare D, Baron MS. A Novel Tri-Component Scheme for Classifying Neuronal Discharge Patterns. Journal of Neuroscience Methods. 2015;239:148–161. doi:10.1016/j.jneumeth.2014.09.015.

44. Mijatović G, Lončar-Turukalo T, Procyk E, Bajić D. A Novel Approach to Probabilistic Characterisation of Neural Firing Patterns. Journal of Neuroscience Methods. 2018;305:67–81. doi:10.1016/j.jneumeth.2018.05.005.

45. Roudi Y, Dunn B, Hertz J. Multi-Neuronal Activity and Functional Connectivity in Cell Assemblies. Current Opinion in Neurobiology. 2015;32:38–44. doi:10.1016/j.conb.2014.10.011.

46. Kobayashi R, Kurita S, Kurth A, Kitano K, Mizuseki K, Diesmann M, et al. Reconstructing Neuronal Circuitry from Parallel Spike Trains. Nature Communications. 2019;10(1):4468. doi:10.1038/s41467-019-12225-2.

47. Shorten DP, Spinney RE, Lizier JT. Estimating Transfer Entropy in Continuous Time between Neural Spike Trains or Other Event-Based Data. PLOS Computational Biology. 2021;17(4):e1008054. doi:10.1371/journal.pcbi.1008054.

48. Stramaglia S, Scagliarini T, Daniels BC, Marinazzo D. Quantifying Dynamical High-Order Interdependencies from the O-information: An Application to Neural Spiking Dynamics. Frontiers in Physiology. 2021;11:595736. doi:10.3389/fphys.2020.595736.

49. Peyrache A, Benchenane K, Khamassi M, Wiener SI, Battaglia FP. Principal Component Analysis of Ensemble Recordings Reveals Cell Assemblies at High Temporal Resolution. Journal of Computational Neuroscience. 2010;29(1):309–325. doi:10.1007/s10827-009-0154-6.

50. Humphries MD. Spike-Train Communities: Finding Groups of Similar Spike Trains. Journal of Neuroscience. 2011;31(6):2321–2336. doi:10.1523/JNEUROSCI.2853-10.2011.

51. Lopes-dos-Santos V, Ribeiro S, Tort ABL. Detecting Cell Assemblies in Large Neuronal Populations. Journal of Neuroscience Methods. 2013;220(2):149–166. doi:10.1016/j.jneumeth.2013.04.010.

52. Billeh YN, Schaub MT, Anastassiou CA, Barahona M, Koch C. Revealing Cell Assemblies at Multiple Levels of Granularity. Journal of Neuroscience Methods. 2014;236:92–106. doi:10.1016/j.jneumeth.2014.08.011.

53. Herzog R, Morales A, Mora S, Araya J, Escobar MJ, Palacios AG, et al. Scalable and Accurate Method for Neuronal Ensemble Detection in Spiking Neural Networks. PLOS ONE. 2021;16(7):e0251647. doi:10.1371/journal.pone.0251647.

54. Gansel K, Singer W. Detecting Multineuronal Temporal Patterns in Parallel Spike Trains. Frontiers in Neuroinformatics. 2012;6. doi:10.3389/fninf.2012.00018.

55. Quaglio P, Rostami V, Torre E, Grün S. Methods for Identification of Spike Patterns in Massively Parallel Spike Trains. Biological Cybernetics. 2018;112(1):57–80. doi:10.1007/s00422-018-0755-0.

56. Mackevicius EL, Bahle AH, Williams AH, Gu S, Denisenko NI, Goldman MS, et al. Unsupervised Discovery of Temporal Sequences in High-Dimensional Datasets, with Applications to Neuroscience. eLife. 2019;8:e38471. doi:10.7554/eLife.38471.

57. Tingley D, Peyrache A. On the Methods for Reactivation and Replay Analysis. Philosophical Transactions of the Royal Society B: Biological Sciences. 2020;375(1799):20190231. doi:10.1098/rstb.2019.0231.

58. Williams AH, Poole B, Maheswaranathan N, Dhawale AK, Fisher T, Wilson CD, et al. Discovering Precise Temporal Patterns in Large-Scale Neural Recordings through Robust and Interpretable Time Warping. Neuron. 2020;105(2):246–259.e8. doi:10.1016/j.neuron.2019.10.020.

59. Sotomayor-Gómez B, Battaglia FP, Vinck M. Spikeship: A Method for Fast, Unsupervised Discovery of High-Dimensional Neural Spiking Patterns. PLOS Computational Biology. 2023;19(7):e1011335. doi:10.1371/journal.pcbi.1011335.

60. Rigotti M, Barak O, Warden MR, Wang XJ, Daw ND, Miller EK, et al. The Importance of Mixed Selectivity in Complex Cognitive Tasks. Nature. 2013;497(7451):585–590. doi:10.1038/nature12160.

61. Bernardi S, Benna MK, Rigotti M, Munuera J, Fusi S, Salzman CD. The Geometry of Abstraction in the Hippocampus and Prefrontal Cortex. Cell. 2020;183(4):954–967.e21. doi:10.1016/j.cell.2020.09.031.
