# Supplementary figures and images for: Synchrony, oscillations, and phase relationships in collective neuronal activity: A highly comparative overview of methods
Source: PLoS Comput Biol. 2025 Oct 24;21(10):e1013597. doi: 10.1371/journal.pcbi.1013597 (PMC12574956; doi:10.1371/journal.pcbi.1013597)

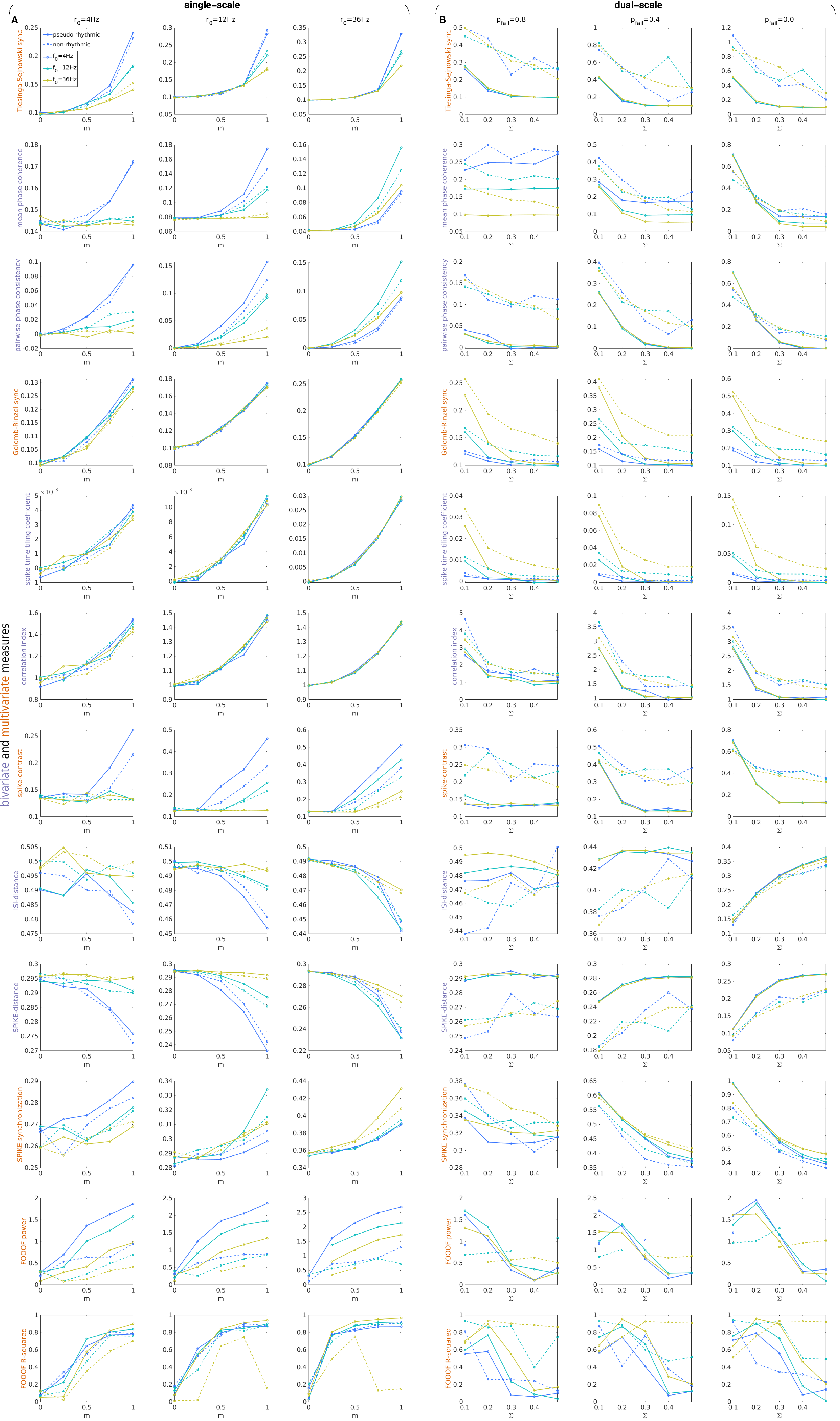

Supplement: S1 Fig — Assessment of the level of synchrony on single-scale (left) and dual-scale (right) synthetic spike trains, considering multiple MSTMs. Left: Synchrony is plotted as a function of the modulation amplitude m for average firing rates r0 and population rates f0 varying in the grid [4,12,36] Hz × [4,12,36] Hz. Results for different average firing rates r0 are plotted in separate columns. Right: Synchrony is plotted as a function of the population width Σ for population rates f0 and spike deletion probability pfail varying in the grid [4,12,36] Hz × [0.8,0.4,0]. Results for different spike deletion probabilities pfail are plotted in separate columns. In all panels, the population rate f0 varies in the grid [4,12,36] Hz and is color-coded, with warmer colors indicating higher f0. Solid lines: pseudo-rhythmic spike trains; dash lines: non-rhythmic spike trains. (PNG) [file pcbi.1013597.s002.png]

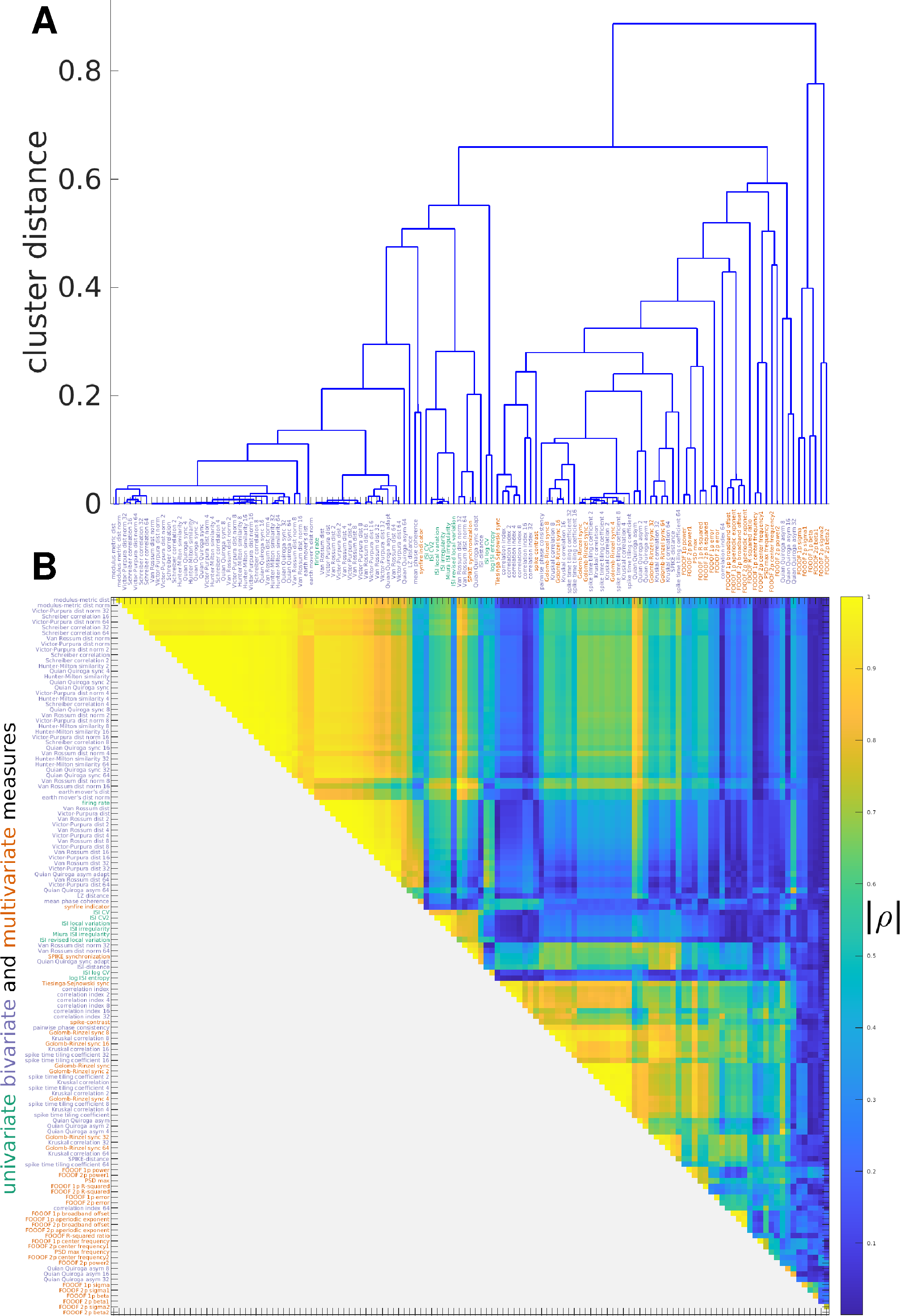

Supplement: S2 Fig — A: Dendrogram showing the distances between MSTMs as a hierarchical cluster tree. Measures are sorted along the x axis in increasing order of the dendrogrammatic distance of the first nonsingleton cluster they are grouped in. B: Similarity matrix showing the absolute value of the Spearman correlation coefficient between each pair of MSTMs. Measure labels are color-coded to indicate measure type (green: univariate; blue: bivariate; red: multivariate). Numbers displayed at the end of timescale-dependent measures indicate the timescale in milliseconds. For FOOOF spectral measures, 1p (2p) refers to the single-peak (dual-peak) model; in the dual-peak model, the number at the end of the parameter name indicates the corresponding peak. (PNG) [file pcbi.1013597.s003.png]

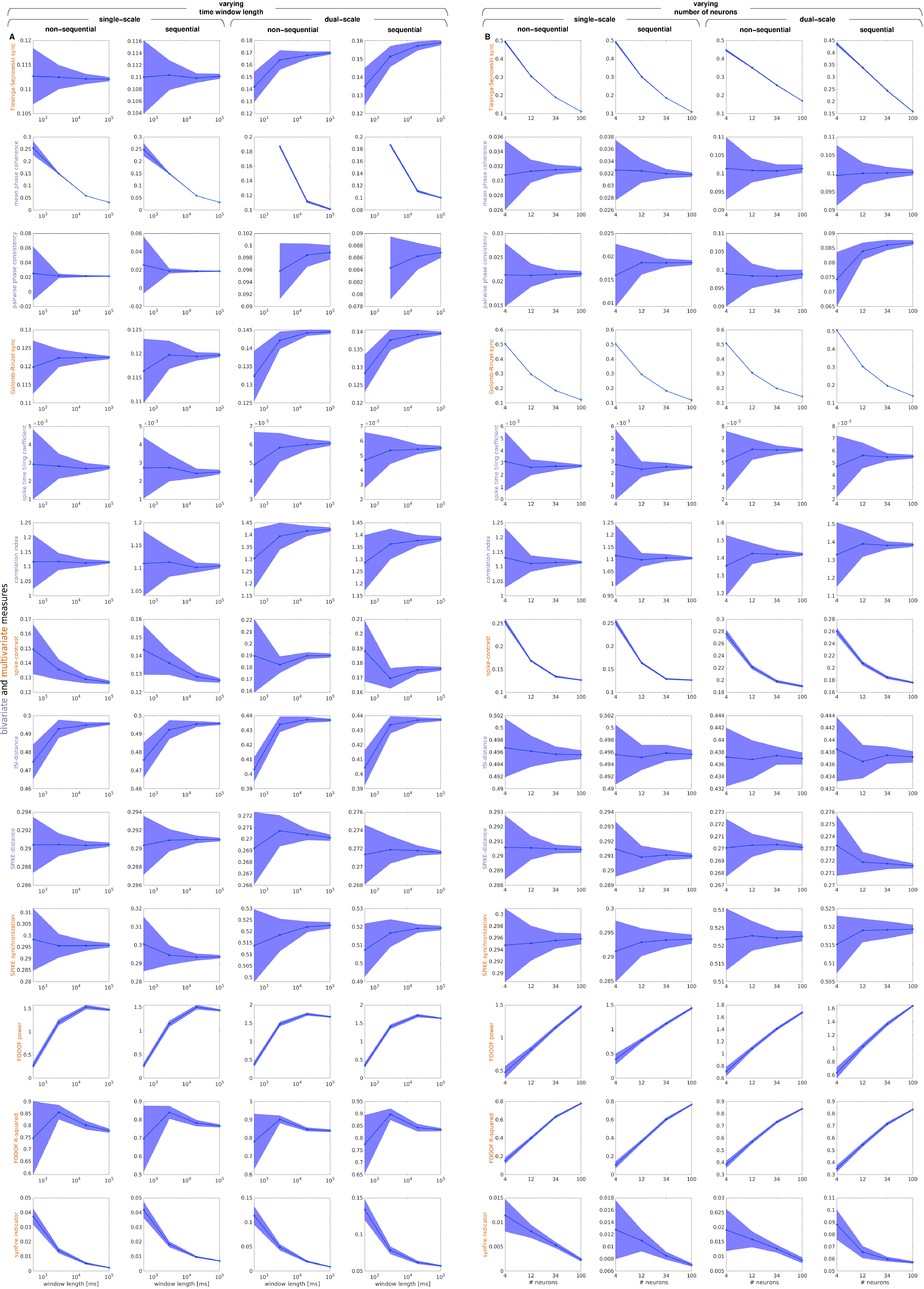

Supplement: S3 Fig — Effects of sample size variations in time (A) or space (B) for a subset of selected MSTMs. A: For each MSTM, the mean value across windows is plotted as a function of window length. Shaded areas indicate the SD across windows. Different columns correspond to different spike train families and generative parameter values: single-scale pseudo-rhythmic spike train with r0=f0=12Hz, m = 0.5 (non-sequential, left; sequential with Dc = 0.2, center-left); dual-scale pseudo-rhythmic spike train with f0=12Hz, Σ=0.2, pfail=0.4, (non-sequential, center-right; sequential with Dc = 0.2, right). MPC and PPC could not be estimated at the shortest time window length considered due to insufficient number of spikes. B: As in (A), but sample size varies in space instead of time. (PNG) [file pcbi.1013597.s004.png]

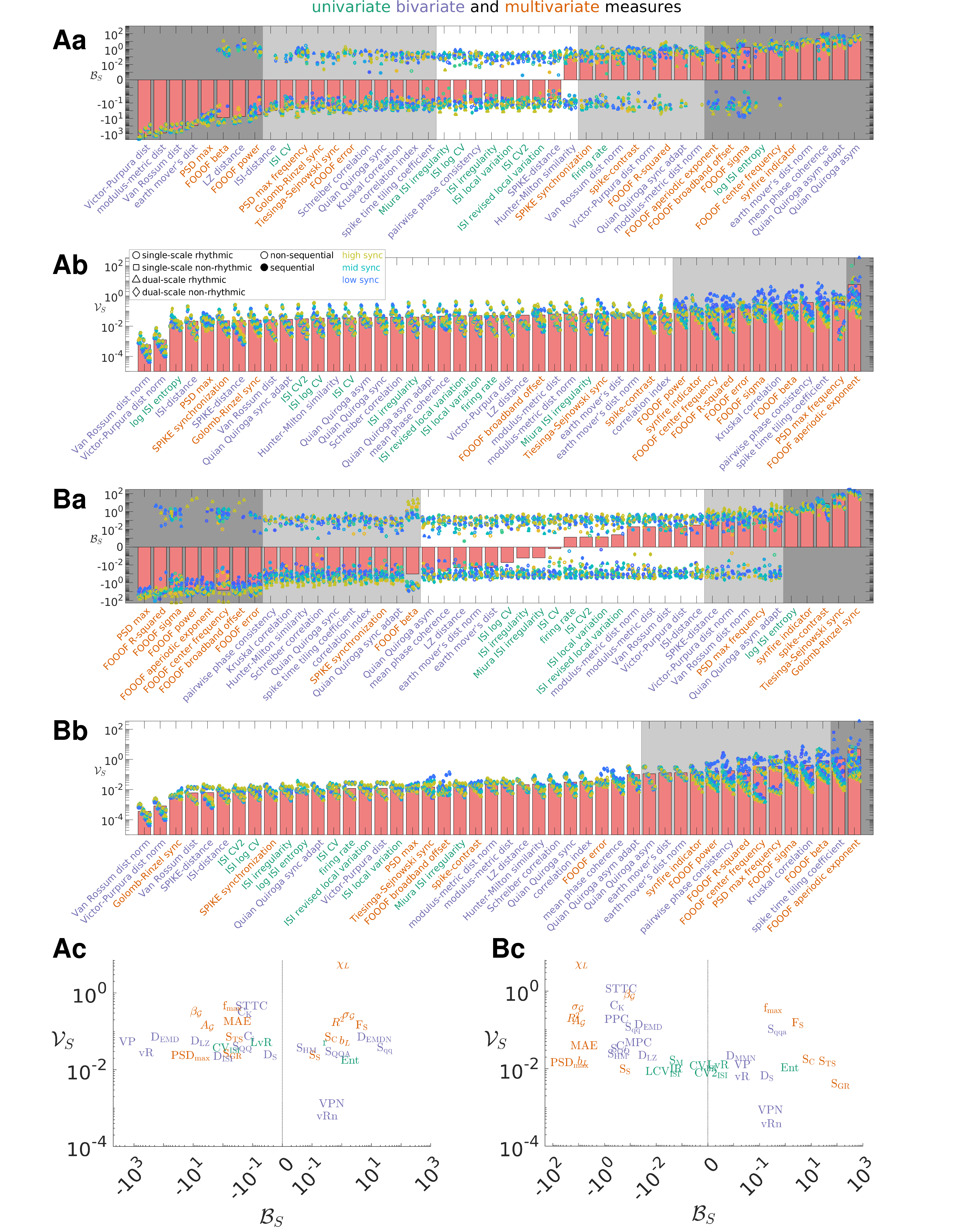

Supplement: S4 Fig — A: Bias and variability resulting from a finite time window length. Measures are ordered accordingly to increasing bias (a) or variability (b). Bars indicate mean values across synthetic spike train families, synchrony values and time window length Twin, with individual values shown by symbols as indicated in the legend. Other parameters are fixed at intermediate values: r0=f0=12Hz for the single-scale family, f0=12Hz and pfail=0.4 for the dual-scale family, Dc = 0 or 0.2 for non-sequential and sequential trains, respectively. Results corresponding to time windows of increasing length are plotted from left to right for each MSTM. Variability is plotted against bias in (c); best behavior corresponds to minimum 𝒱S and |ℬS|. Some measures are omitted for clarity. In (a), the gray background indicates MSTMs with bias |ℬS| > 0.1 (light gray) or |ℬS| > 1 (dark gray). In (b), the gray background indicates MSTMs with variability 𝒱S > 0.1 (light gray) or 𝒱S > 1 (dark gray). Note the use of a symmetric log scale for bias in (a) and (c). B: As in (A), but sample size varies in space instead of time. Corresponding results are plotted for each individual Twin (Nneu) value separately in S5 Fig (S6). This figure conveys information on temporal and spatial finite-size effects at multiple levels of detail, from average values (bars) to individual results for each spike train formalism (symbols), with symbol color coding for the level of synchrony and symbol type coding for spike train formalism. (PNG) [file pcbi.1013597.s005.png]

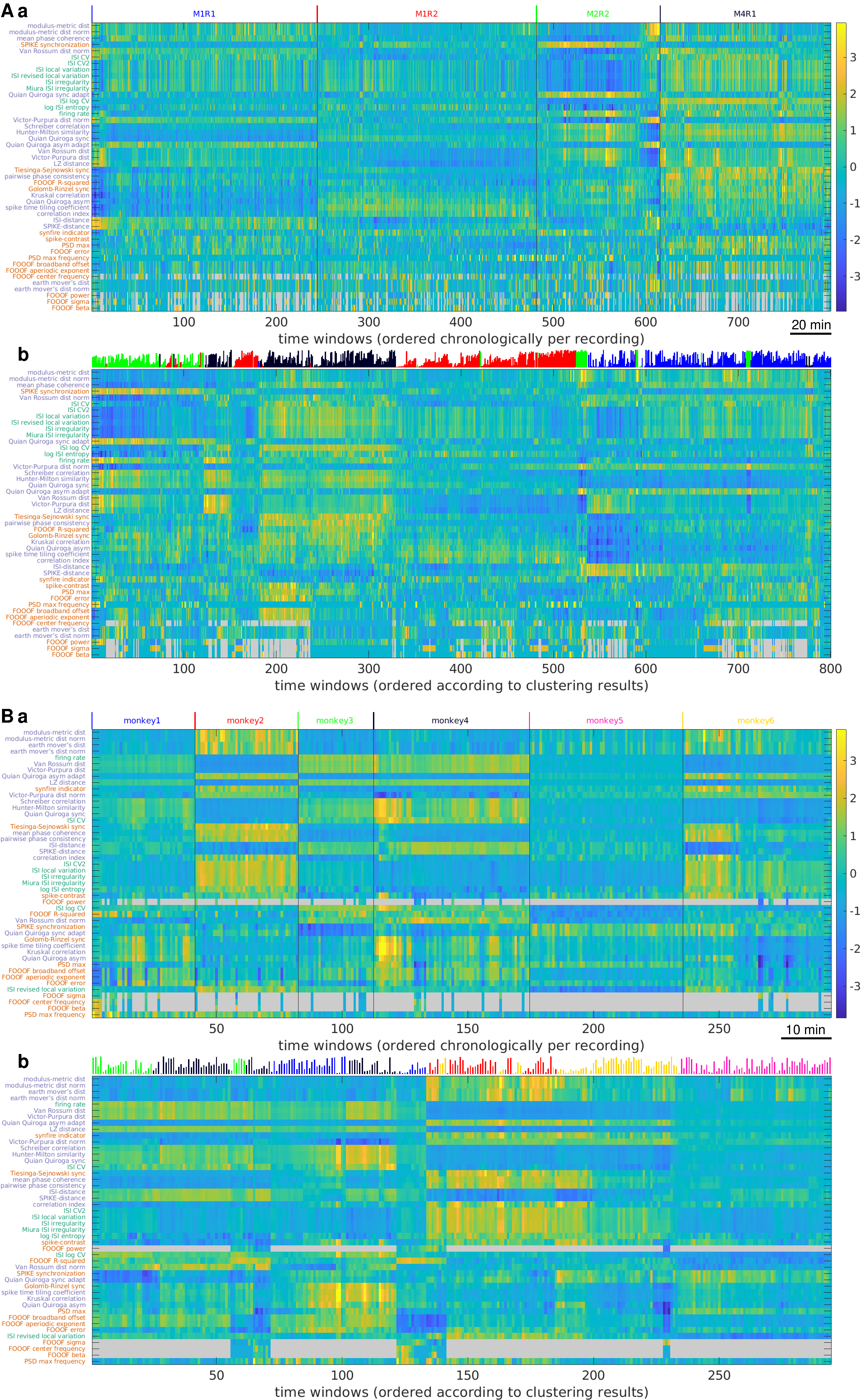

Supplement: S7 Fig — As in Fig 6, for the mouse (A) and monkey (B) datasets. (PNG) [file pcbi.1013597.s008.png]

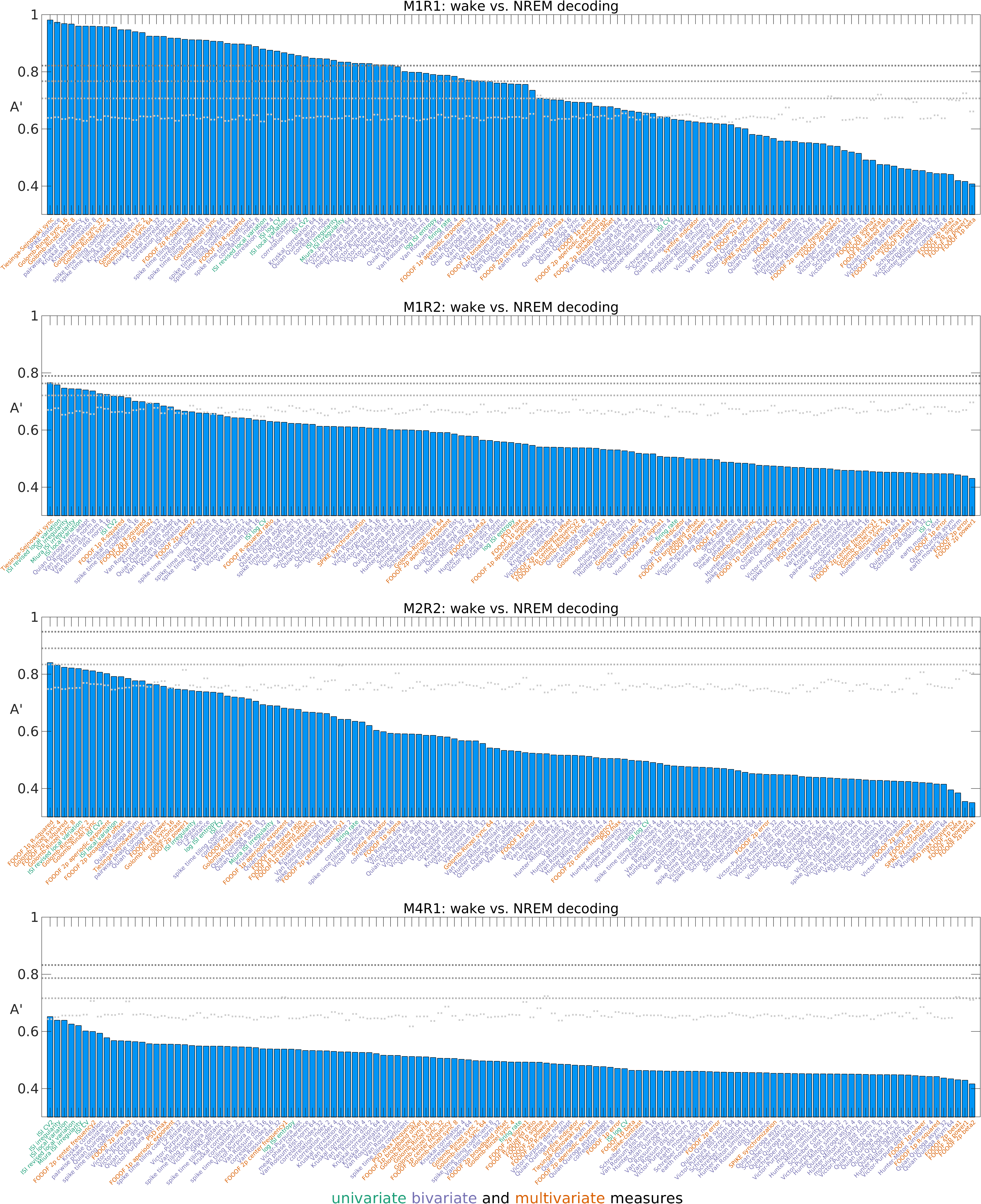

Supplement: S9 Fig — As in Fig 8A, for each individual recording and for each MSTM of the extended set of 131 MSTMs. (PNG) [file pcbi.1013597.s010.png]

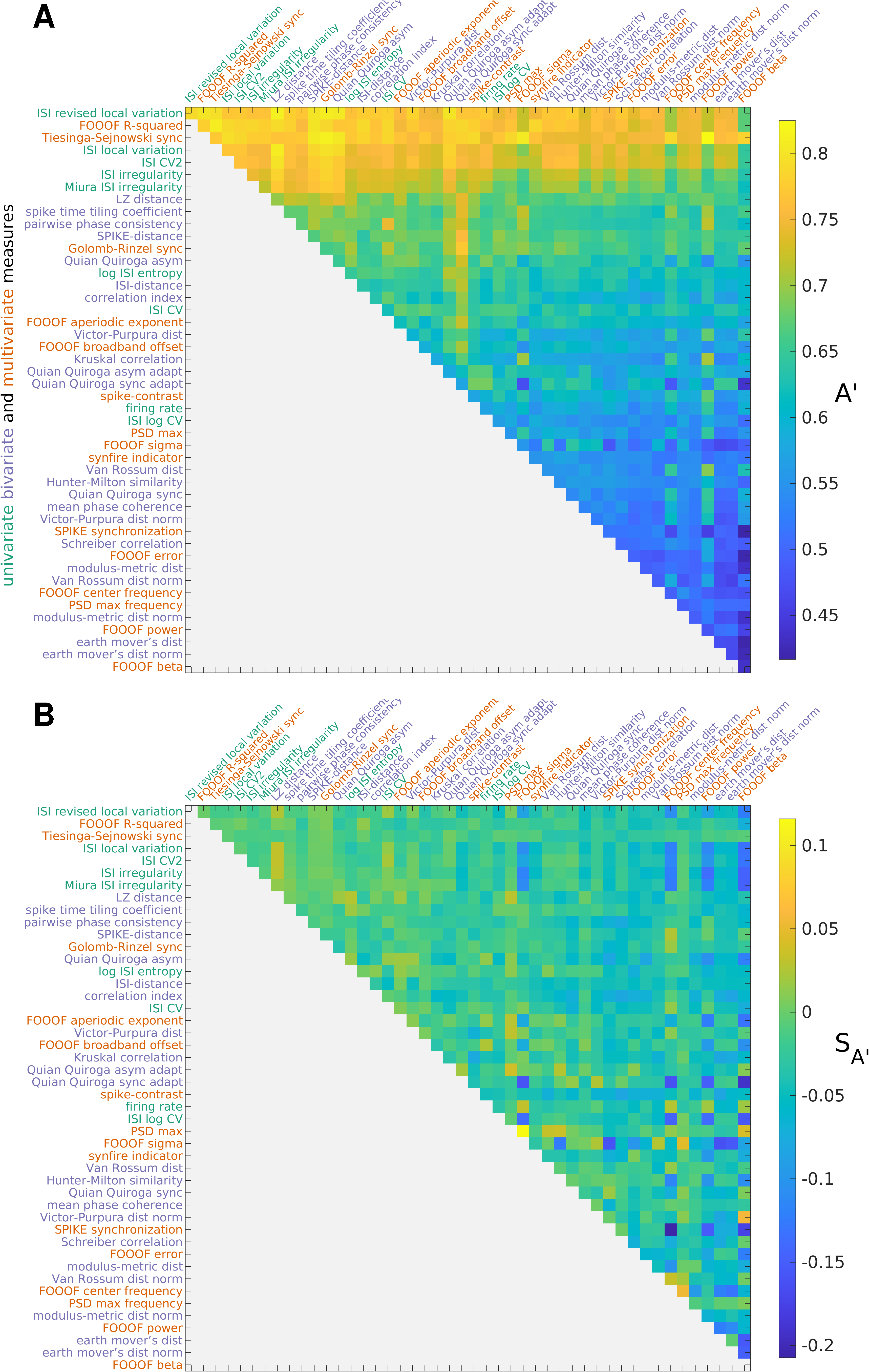

Supplement: S11 Fig — As in Fig 9, but median results across recordings are shown. (PNG) [file pcbi.1013597.s012.png]

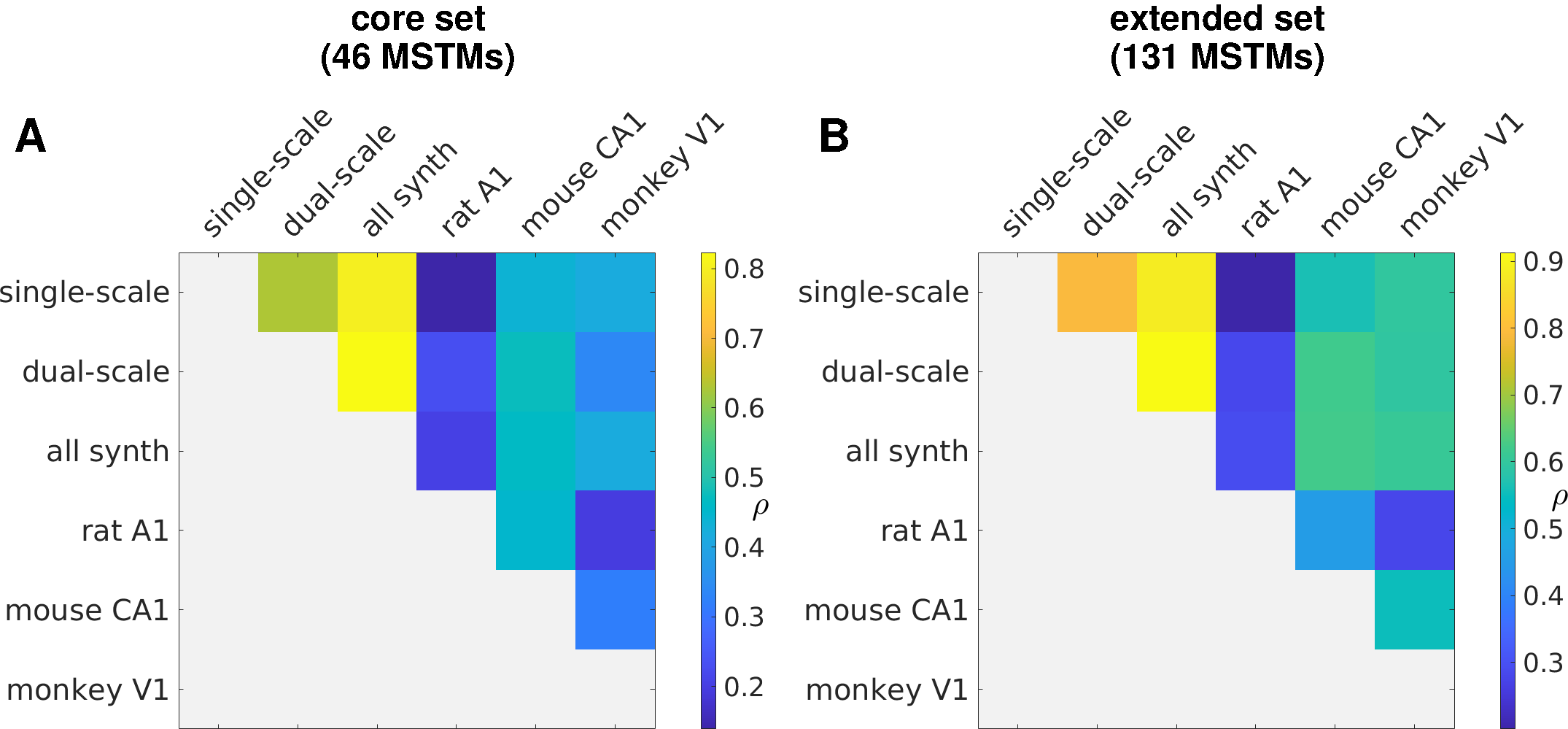

Supplement: S12 Fig — Correlation between pairs of inter-MSTM distance matrices obtained from synthetic spike train families and biological spike train datasets considering either the core set of 46 MSTMs (A) or the full set of 131 MSTMs (B). (PNG) [file pcbi.1013597.s013.png]
