# Supplementary material for: Synchrony, oscillations, and phase relationships in collective neuronal activity: A highly comparative overview of methods
Source: PLoS Comput Biol. 2025 Oct 24;21(10):e1013597. doi: 10.1371/journal.pcbi.1013597 (PMC12574956; doi:10.1371/journal.pcbi.1013597)
Supplement: Attachment — Submitted filename: sync_osc_ms_response.pdf [file pcbi.1013597.s016.pdf]

Response to Editorial Assessment

August 15th 2024

Dear Dr. Bush and Dr. Serre,

We would like to submit a new version of our manuscript "Synchrony, oscillations, and phase relationships in collective neuronal activity: a highly-comparative overview of methods".

The manuscript has been considerably shortened, according to your suggestion. In particolar, the most general Methods subsections have been moved to the Supplementary Methods. Also, parts of the Discussion have been moved to the Supplementary Discussion, and the last figure of the previous version is now included as a Supplementary Figure.

Overall, the main manuscript is now less than 54 pages long (including references), which corresponds to a reduction of $\sim 15\%$ with respect to its previous length of 63 pages. The Discussion section now amounts to less than 4 pages. The number of references was also reduced from 150 to 115, with some references (i.e., those related to general methods, or to potential future directions that are not within the actual scope of the article) now included only in the Supplementary Information.

We believe that these modifications yielded a more succinct description of our results and message, while still including the most important components in the main manuscript.

For the convenience of reviewers and readers, the Supplementary Information is also available as a single pdf file at this link, which is included at the start of the Supporting information subsection. The code is available at `https://github.com/fabaroni/sync_osc_MSTMs`.

Yours sincerely,

Fabiano Baroni & Ben Fulcher
