# Supplementary material for: Synchrony, oscillations, and phase relationships in collective neuronal activity: A highly comparative overview of methods
Source: PLoS Comput Biol. 2025 Oct 24;21(10):e1013597. doi: 10.1371/journal.pcbi.1013597 (PMC12574956; doi:10.1371/journal.pcbi.1013597)
Supplement: Attachment — Submitted filename: sync_osc_ms_rebuttal.pdf [file pcbi.1013597.s017.pdf]

March 11th 2025

Dear Editors,

We acknowledge the useful report we received, which greatly helped to improve our manuscript. In the following, we address the Editors' and Reviewers' concerns in detail, and indicate the corresponding changes that have been made to our manuscript (where applicable).

Answer to the Editors' Comments

The Editors wrote:
"The authors should endeavour to simplify the manuscript and remove as much superfluous detail as possible. The same goes for the supporting code, which must be clear and concise if it is to be of benefit to the community. In particular, they must endeavour to draw clear conclusions and make clear recommendations from this survey of phase coupling methods – which metric, or small number of metrics, would they recommend using in most common experimental contexts, and what are the most important caveats or assumptions that might influence this decision?"

Answer:
Reviewer 1 found the manuscript too long and suggested the removal of the short paragraphs at the start of the Methods and Results section (lines 1360–1364). Removing those paragraphs would result in only a modest reduction of the overall manuscript length, and we find those paragraphs useful for easier orientation and navigation through the manuscript. Hence, those paragraphs were retained in this revised version of the manuscript.

We also acknowledge that Reviewer 2 suggested the inclusion of additional synthetic spike train formalisms (lines 1542–1553). Hence, while some readers might find the current manuscript too long, other readers would like to see it covering more material, suggesting that the material currently included achieves an appropriate balance between breadth of scope and manuscript length for most readers. In particular, we believe that the manuscript cannot be reduced further without compromising its scientific quality.

A discussion on the top-performing synchrony MSTMs, with their strengths and weaknesses as identified from the analyses on synthetic spike trains presented in subsections "Effects of synchrony and oscillations" and "Effects of time window length and number of neurons", is included in the subsection "Comparison between MSTMs' behavior on synthetic spike trains" in the Supplementary Discussion in Text S1. We clarified and extended that part, which now reads as follows (please note that reference numbers correspond to the Supplementary Bibliography):

"Overall, the MSTM that most closely correlates with synchrony is the Correlation index $\mathrm{C_i}$ [19] (Fig 3). This MSTM is only moderately affected by pseudo-rhythmic oscillations and is relatively insensitive to potential confounds related to differences in firing rate or population frequency. It also exhibits little temporal finite-size bias, but it is not among the best MSTMs in terms of temporal finite-size precision and spatial finite-size effects.

A measure derived from the FOOOF spectral estimation, FOOOF power ($A_{\mathcal{G}}$), achieves a high correlation with the parameter determining synchrony in dual-scale trains. However, a numerical value for that MSTM cannot be obtained for every spike train, since a spectral peak with an amplitude exceeding a certain threshold is required, as explained in the Methods subsection "Multineuron spike train measures".

Tiesinga–Sejnowski synchrony S_{TS} is also closely correlated with the parameter determining synchrony for all synthetic spike train families (Fig 3). However, it is highly sensitive to pseudo-rhythmic oscillations, especially for the dual-scale synthetic families. It is worth noting that S_{TS} markedly decreases with oscillations in dual-scale synthetic spike trains, while it is relatively unaffected (or can even display small increases, depending on parameter values) in single-scale spike trains (Fig 2 and S1), indicating that oscillations affect this MSTM in a way that depends on the specific structure of the synthetic spike train families considered.

Another measure that achieves good performance is the spike time tiling coefficient STTC [20]. This MSTM correlates closely and relatively selectively with synchrony in both single-scale and dual-scale trains, and is only modestly biased when varying T_{win} or N_{neu}. However, it is highly variable across different realizations of the same generative model (Fig 6, Ab and Bb).

Other MSTMs that perform relatively well are Golomb–Rinzel synchrony S_{GR} [21], pairwise phase consistency PPC [22], and Kruskal correlation C_K [23]. These measures exhibit relatively high and selective correlation with synchrony, but they are not among the best measures in terms of bias. It is worth noting that PPC is a measure of phase consistency irrespective of lag, and will hence yield a high value also in the case of consistent phase relationships with non-zero lag. As expected from a phase-based measure, it is sensitive to pseudo-rhythmicity in dual-scale trains."

We also updated the code repository following the recommendations from Reviewer 2, which improved its usability (lines 1723–1753, 1769–1833).

Answer to Reviewer 1 (Stephen Eglen)

Reviewer 1 wrote:
"I found the paper "Synchrony, oscillations, and phase relationships in collective neuronal activity: a highly comparative overview of methods" to be a impressive effort to screen a large range of bivariate methods (and some univariate methods) for assessing synchrony of various forms in neural spike trains.

The authors have clearly spent a lot of time implementing key methods that are freely available, and as an open science project, this is first-class. The paper is very clearly within the remit of PLOS Comp Bio, and I would be supportive of publication. I do however have some concerns that I would like the authors to consider.

As a declaration of interest, I co-wrote a similar paper with Catherine Cutts in 2014 (citation 65 in the paper). I am very supportive of such approaches, and the general philosophy in the paper. It is by doing independent comparative studies of methods that we can understand a richer understanding of neural dynamics. A key strength here I feel is the first part of the paper where synthetic ground-truth data shows the pros/cons of each method."

Answer:
We thank Dr. Eglen for his supportive comments.

Reviewer 1 wrote:
"Major concerns

It might just be my personal experience, but quite often scientists often want to be told which method they should use for a particular dataset. My key take-home from reading the paper is that, unsurprisingly, there is no 'best method' and we need to be careful in understanding the strengths and limitations of each method. My biggest concern about the paper is that it does not really narrow down the selection of methods."

Answer:
We thank the Reviewer for his observation. We agree that many readers would appreciate a selection of "best methods" that could be employed for most applications.

A discussion on the top-performing synchrony MSTMs, with their strengths and weaknesses as identified from the analyses on synthetic spike trains presented in subsections "Effects of synchrony and oscillations" and "Effects of time window length and number of neurons", is included in the subsection "Comparison between MSTMs' behavior on synthetic spike trains" in the Supplementary Discussion in Text S1. We clarified and extended that part, which now reads as follows (please note that reference numbers correspond to the Supplementary Bibliography):

"Overall, the MSTM that most closely correlates with synchrony is the Correlation index $\mathrm{C_i}$ [19] (Fig 3). This MSTM is only moderately affected by pseudo-rhythmic oscillations and is relatively insensitive to potential confounds related to differences in firing rate or population frequency. It also exhibits little temporal finite-size bias, but it is not among the best MSTMs in terms of temporal finite-size precision and spatial finite-size effects.

A measure derived from the FOOOF spectral estimation, FOOOF power ($A_{\mathcal{G}}$), achieves a high correlation with the parameter determining synchrony in dual-scale trains. However, a numerical value for that MSTM cannot be obtained for every spike train, since a spectral peak with an amplitude exceeding a certain threshold is required, as explained in the Methods subsection "Multineuron spike train measures".

Tiesinga–Sejnowski synchrony S_{TS} is also closely correlated with the parameter determining synchrony for all synthetic spike train families (Fig 3). However, it is highly sensitive to pseudo-rhythmic oscillations, especially for the dual-scale synthetic families. It is worth noting that S_{TS} markedly decreases with oscillations in dual-scale synthetic spike trains, while it is relatively unaffected (or can even display small increases, depending on parameter values) in single-scale spike trains (Fig 2 and S1), indicating that oscillations affect this MSTM in a way that depends on the specific structure of the synthetic spike train families considered.

Another measure that achieves good performance is the spike time tiling coefficient STTC [20]. This MSTM correlates closely and relatively selectively with synchrony in both single-scale and dual-scale trains, and is only modestly biased when varying T_{win} or N_{neu}. However, it is highly variable across different realizations of the same generative model (Fig 6, Ab and Bb).

Other MSTMs that perform relatively well are Golomb–Rinzel synchrony S_{GR} [21], pairwise phase consistency PPC [22], and Kruskal correlation C_K [23]. These measures exhibit relatively high and selective correlation with synchrony, but they are not among the best measures in terms of bias. It is worth noting that PPC is a measure of phase consistency irrespective of lag, and will hence yield a high value also in the case of consistent phase relationships with non-zero lag. As expected from a phase-based measure, it is sensitive to pseudo-rhythmicity in dual-scale trains."

Reviewer 1 wrote:
"By contrast, in our 2014 study, we explicitly showed the problems with earlier methods, e.g. Correlation index [citation 66] and why it shouldn't be used. I was surprised to see that correlation index was one of the measures used; a key problem being is that it is vastly different from most other methods; anticorrelated spike trains have indices in the range [0,1]; 1 is the expectation for uncorrelated spike trains; [1, inf] is the range for correlated spike trains. The authors (eq 13 and 14 on page 9) corrected the T-S method along similar lines, so why not try to correct the correlation index, or (preferably), drop it?"

Answer:
The 2014 article by Cutts & Eglen has actually been one of our main sources of inspiration, and we included their code in our work. However, here we adopted a different approach. In particular, we refrained from narrowing down the set of acceptable methods based on their behavior in specific cases (such as such as perfectly correlated or anticorrelated trains, or completely uncorrelated trains), but opted for a more data-driven approach, whereby methods are evaluated and organized based on their empirical behavior (on both synthetic and real spike trains) alone.

Our conceptual framework is expressed in the Introduction:

"Here, we address these challenges by compiling a large battery of statistical measures and applying them to a large set of synthetic and biological spike trains in a systematic fashion, adopting a highly comparative methodology. In the last decade, the highly comparative approach has emerged as a powerful tool for unveiling the empirical structure of analysis methods in a systematic and data-driven manner [26]."

"The results can recapitulate known theoretical relationships between methods using a data-driven approach, and can also offer unexpected insights into both the methods and the datasets being analyzed. For example, they can reveal surprising similarities in the behavior of different analysis methods that could not be inferred from their formal definition. A systematic empirical comparison between statistical measures is required to obtain a broad and general understanding of their behavior, since theoretical relationships can be established only in very few cases and under specific assumptions (e.g., [30])."

In particular, we opted for not modifying existing measures unless a straightforward modification could clearly improve an existing measure, and such a modification—or a similar one—was not already included in some other measure of the MSTM set. Based on these criteria, only the S_{TS} measure was susceptible to being modified.

It is also worth noting that, in most applications, the way a MSTM output varies depending on changes in spike train synchrony (or some other feature) is more important than its absolute value for particular spike trains. That is the case in the common scenario where MSTM outputs for different experimental conditions are statistically compared. Most of our analyses are based on Spearman correlation values, which are not sensitive to MSTM output values but only to how they vary in different conditions (e.g. for different values of the generative parameters as shown in Fig 3, where the Correlation index performs comparatively well).

To clarify our approach, in this revised version we added the following paragraph at the end of subsection "Comparison between MSTMs' behavior on synthetic spike trains" in the Supplementary Discussion in Text S1 (reference numbers correspond to the Supplementary Bibliography):

"In our approach, MSTMs are evaluated based on how their output values change in response to changes in the generative parameters. We believe that this is the most important MSTM property for experimental applications, which often involve the assessment of statistical differences between conditions. In particular, we did not consider absolute MSTM output values. We note, however, that other authors designed or evaluated MSTMs based also on the expected numeric values they yield for reference spike trains, such as perfectly correlated or anticorrelated trains, or completely random trains [20–22,29–31,34–36]."

We also note that presenting a substantial modification of an existing measure (to the point that it could be considered as a novel measure) might not align well with the philosophy of the Benchmarking Collection, as stated in the 2018 editorial by Peters et al. (https://doi.org/10.1371/journal.pcbi.1006494):

"Novel tools created by the authors in parallel with the benchmark should not be included in the article, as they are essentially guaranteed to perform well."

Reviewer 1 wrote:

"The paper currently has the feel of a high-throughput approach analysing all data with all methods. Whilst I see the benefits of this in some contexts, I struggled to see what I could learn from it. It would be very helpful if the cases studies from neural data sets could be used to show the particular strengths of key methods; this could in turn be used to make recommendations."

Answer:

We thank the Reviewer for his suggestion. The strengths and weaknesses of the various methods are evaluated and compared mostly in the first part of the article using synthetic spike trains with known ground truth.

In the second part, using biological spike trains, there is a subsection that evaluates and compares MSTMs (both individually and in pairs) with respect to the information they bear for discriminating between wake and NREM sleep using spiking data from the mouse hippocampus. These analyses are presented as a demonstration of how a highly comparative approach can be leveraged to identify the most informative MSTM or MSTM combination for a particular application, and the types of MSTM pairs that provide synergistic or redundant information.

While some of the top-performing MSTMs in the decoding analyses also performed well with synthetic spike trains (e.g., as in the case of Tiesinga–Sejnowski synchrony S_{TS}), we believe that the results on synthetic spike trains are more relevant for providing general recommendations. While it is likely that the

decoding results we provide might generalize to other datasets, it is less likely that they will generalize to the discrimination between other behavioral states, or between different stimulation conditions. On the other hand, the results with synthetic spike trains are more general, since we adopted two different synthetic spike train generative models, both based on simple mathematical formalisms.

However, the decoding results using the mouse dataset are also useful for illustrating how a highly comparative approach, including both synchrony or oscillation MSTMs as well as univariate MSTMs of firing intensity and variability, can be used to gauge the relative discriminability of these various MSTMs (both within and between MSTM types), as commented in the Discussion:

"We also illustrated how this approach can be leveraged for supervised classification, in particular for the estimation of recording-wise distinctiveness (Fig 8) and for discriminating between awake and NREM sleep (Fig 9, 10, and 11, and Figs S8, S9 and S10). Among the most discriminating MSTMs for the latter, we found some MSTM that has already been emphasized as displaying a high correlation with the parameter determining synchrony in synthetic spike trains, such as S_{TS}. However, we also found univariate measures of instantaneous ISI variability such as LvR, and measures of spectral fitting quality such as the FOOOF R-squared, which are rarely considered in studies on neuronal synchrony and oscillations. In fact, among the top 7 MSTMs at the population level, 5 of them are univariate measures of instantaneous ISI variability. This is a surprising result, given that variations in consciousness state are thought to be mediated by changes in collective neuronal coordination patterns. Other univariate measures yielded only marginally significant results (as in the case of CV_{ISI}, a classic measure of global ISI variability which is sensitive to nonstationarity and firing rate) or non-significant results (as in the case of the mean firing rate r). This further emphasizes the utility of adopting a highly comparative approach where multiple MSTMs at various levels of description are contrasted. Among the bivariate and multivariate measures, the most discriminative ones do not correspond with those that exhibit the most selective correlation with the parameter determining synchrony in synthetic spike trains (Fig 3), but tend to capture a combination of features. For example, S_{TS} is sensitive to both synchrony and pseudo-rhythmic properties of input trains. Hence, while measure selectivity is surely a desirable property, and chiefly so if interpretability is key, it might not be a decisive factor if decoding accuracy is the main objective."

The application of our MSTM library to biological datasets also serves to illustrate how our approach can be used for quantifying recording-wise distinctiveness and its dependence on the dimensionality of the spike train representation (Fig 8 and Table 4), and for estimating the intrinsic dimensionality of the MSTM library in relation to the corresponding results obtained with synthetic spike trains (Fig 12). The results of these analyses motivated one of the central messages of our article:

"These results suggest a view of spike train coordination as a multidimensional concept that is best quantified by considering a battery of MSTMs, instead of one or a few measures, preselected with only minimal comparison to potential alternatives."

Reviewer 1 wrote:
"Minor concerns

The abstract is missing what I think is the key conclusion – there is no 'single best measure'."

Answer:
We thank the Reviewer for the suggestion. In this version, that is included in the Author Summary, specifically in the sentence included below:

"Our analyses indicate that there is no overall "best" measure, and inform on the relative advantages and drawbacks of a broad range of measures with respect to several criteria of interest for their empirical application, ..."

Including that sentence in the Abstract would also be a good choice, but having it in the Author Summary also seems a good (and perhaps slightly better) option, since the Author Summary is meant to provide an even more concise description of the study, targeted to a broader audience and conveying its significance also to readers who may not have specialized expertise in the field.

We also note that the absence of references to any specific measure in the Abstract implicitly suggests that there is no overall best measure.

Reviewer 1 wrote:

"Many of the methods contain parameters (e.g. for timescale) – how did you test the effect of these parameters."

Answer:

Indeed, many MSTMs include a timescale parameter that determines the temporal range defining synchrony. We considered both a core set of MSTMs, where the timescale parameter was fixed at 1 ms, and an extended set of MSTMs, where the timescale parameter was varied in a range, as stated in the Methods subsection "Multineuron spike train measures":

"We also extended this core set by including a range of timescales for the timescale-dependent measures, yielding a library of 131 MSTMs, which we refer to as the extended set. Specifically, we considered 7 timescales logarithmically spaced between 1 and 64 ms."

Spectral measures derive from a multi-taper power spectrum estimation. In this revised version, we justify the choice of the multi-taper spectral estimation parameters (i.e., time-bandwidth product and number of tapers) as follows:

"The power spectrum was estimated using a multi-taper method [51] implemented in the Chronux data analysis toolbox with a time-bandwidth product of 10 and 19 tapers, resulting in a relatively smooth power estimation that is suitable for spectral feature extraction and parameterization."

FOOOF spectral parameterization requires setting a threshold for peak detection. In this revised version, we justify the choice of the threshold value as follows:

"The threshold for a peak to be included in the model was set to 3 standard deviations (SD) of the residuals after subtraction of the aperiodic component. This threshold is more conservative than the default threshold of 2 SD included in the original FOOOF code, but resulted in the detection of at least one spectral peak in a similar number of cases when applied to synthetic spike trains. Peaks detected only at the 2 SD threshold often appeared spurious upon visual inspection, suggesting that the 3 SD threshold provides a better balance between identifying meaningful peaks and excluding false positives."

Reviewer 1 wrote:

"I didn't understand the 'p hacking comment (2nd para of page 3). It would seem to me on the surface that uncritical application of a large number of methods might lead to p-hacking type behaviour – reporting the one method that produces a desired outcome."

Answer:

The Reviewer is correct in stating that the application of a large number of methods and the selective reporting of the one method that produces a desired outcome would constitute an example of p-hacking. The critical contribution of the highly comparative approach is that a large number of methods are applied and their outcomes are reported, as stated in the Introduction:

"Typically, explorative studies consider a number of statistical measures that are thought to be relevant for the characterization of the processes under examination. Then, it is not uncommon that only those that "worked" (e.g., by exhibiting a statistically significant difference between groups) are reported in scientific publications. By contrast, in the highly comparative approach a sheer number of methods are employed and reported, hence greatly ameliorating publication biases and related issues such as p-hacking."

Reviewer 1 wrote:

"By looking at the letter to the editor, I'm assuming this is a revision of a manuscript, and that in the previous round of reviews the paper was judged to be overly long. It still feels very long; one possibility for cutting it would be drop one of the case studies? Some of the introductory paragraphs (first para of Methods, page 3; bottom para of page 8; first results par,; page 14)"

Answer:

Indeed, in a previous version of the manuscript (available in bioRxiv) the Supplementary Methods and the Supplementary Discussion were included in the main manuscript. The bioRxiv version itself derived from a longer previous version, which also included additional synthetic spike train formalisms where neuronal heterogeneity was varied parametrically. We decided to remove that part and present it in a separate article. However, Reviewer 2 suggested the inclusion of additional synthetic spike train formalisms, specifically proposing the inclusion of spike train generative models that are suitable for the exploration of how neuronal heterogeneity affects MSTMs' behavior (lines 1542–1553 below). Hence, while we acknowledge that some readers might find the current manuscript too long, some other readers would like to see it covering more material. Overall, we believe that the material currently included achieves an appropriate balance between breadth of scope (which, in the case of a highly comparative project, is necessarily wider than average) and manuscript length.

We would prefer to retain all 3 biological spike train datasets. The rat auditory cortex dataset [91, 92] is the only one with a relatively large number of recordings (14 versus 4 in the mouse dataset and 6 in the monkey dataset), enabling the qualitative (i.e. visual) and quantitative assessments of within- and across-recording variability (Fig 7B and Fig 8). The mouse hippocampal dataset [93, 94] is the only one that includes multiple conditions (i.e. wake and NREM sleep), enabling supervised classification (Fig 9, 10, and 11). The monkey visual cortex dataset provides an additional benchmark for illustrating the utility of the highly comparative approach in the various scenarios considered (i.e. for the visualization and organization of real spike trains, and for the quantification of recording-wise distinctiveness) and for the estimation of the MSTM library dimensionality in a biological dataset, and extends our approach to the case of primate cortical spike trains. The removal of the monkey dataset would result in only a modest reduction of the Supplementary Material (i.e. removal of one row in Table 1 in the Supplementary Methods and of the corresponding paragraph in subsection "Biological spike train preprocessing", as well as of panels B of Fig S6 and Fig S7), with hardly any reduction of the main manuscript.

The paragraphs mentioned by the Reviewer were added at the start of the Methods and Results section to orient readers by providing a concise description of what is about to be presented. Removing these short paragraphs would only modestly reduce the overall manuscript length (by less than half a page). We would prefer to retain them, since we believe that these paragraphs help readers' orientation and navigation through what is admittedly a longer-than-average manuscript.

Reviewer 1 wrote:
"FOOOF – I had no idea what this method was. At the very least I would call it 'spectral parameterization' as this is much more understandable to a wider audience. Ordinarily, I think it might be worth a clearer description of what the technique does in the paper; however, given the length of the paper already, I do not think it critical."

Answer:
We acknowledge the Reviewer's concern. While FOOOF (fitting oscillations & one over f) parameterization has become quite popular in the field of M/EEG analysis, it is less widely employed in research at the level of MUA or single neurons.

A description of FOOOF parameterization is reported in the Methods subsection "Multineuron spike train measures":

"We also performed a FOOOF (fitting oscillations & one over f) parameterization of the power spectrum of the instantaneous population firing rate [57], whereby the log power spectrum PSD was described by the sum of an aperiodic component and one or more periodic components:

$$\mathrm{PSD}(f) = L(f) + \sum_{n=0}^{N_p} G_n \,, \tag{20}$$

where $L(f)$ represents the aperiodic component, described as a power-law function with an offset

$$L(f) = b_L - \log f^{\chi_L} \,, \tag{21}$$

and each G_n is a Gaussian fit to a peak:

$$\mathcal{G}(f) = A_{\mathcal{G}} \mathrm{e}^{-\frac{1}{2}\left(\frac{f - f_{\mathcal{G}}}{\sigma_{\mathcal{G}}} \right)^2} . \tag{22}$$

"

We prefer to retain the FOOOF acronym since several different options for spectral parameterization have been proposed (some of which are cited in the manuscript, as in the case of [58–60]), and FOOOF parameterization is one of the few options that fit the broadband aperiodic component and the spectral peaks separately. However, we followed the Reviewer's suggestion and referred to "FOOOF spectral parameterization" wherever there could be a potential ambiguity about FOOOF being a spectral parameterization method. In particular, the following lines have been updated:

"These include measures derived from the FOOOF spectral analysis" [page 19, line 491]

"R^2 (a measure of FOOOF spectral fitting quality)" [page 31, line 630]

"For FOOOF spectral measures" [captions of Fig 11 and Fig S2]

"measures of spectral fitting quality such as the FOOOF R-squared" [page 41, line 856]

Reviewer 1 wrote:
"Fig 3 (and elsewhere); red/green/blue is used to classify measures; I found it very difficult to remember which is which, and simply relied on the measure name, rather than the colour of the text. Is the colour necessary? If you keep colour, bear in mind red/green colourblind. How about just using appending some text (1) (2) (n) to each name to indicate univariate, bivariate and multivariate?"

Answer:

We prefer to use a color code for indicating univariate, bivariate, and multivariate measures, rather than text subscripts or superscripts, since double subscripts are already in use for some measures (such as $f_{\mathcal{G},2\mathrm{p}1}$ and other FOOOF spectral measures), as well as simultaneous subscript & superscript (as in the case of $R^2_{2\mathrm{p}}$), and the inclusion of additional subscripts or superscripts could result in a heavier and less readable notation.

The red and blue colors we use are the canonical red and blue (RGB codes [1 0 0] and [0 0 1], respectively). The green color we use has a blue component that helps differentiation with red by protanopes and deuteranopes (RGB code [0.1961 0.8039 0.1961]).

We considered adopting a slightly different color coding, using colors suggested in the Color Universal Design (CUD) palette (https://jfly.uni-koeln.de/color/image/pallete.jpg):

- Vermillion (RGB: [0.8 0.4 0])
- Bluish green (RGB: [0 0.6 0.5])
- Blue (RGB: [0 0.45 0.7])

We used a color blindness simulator (Coblis, https://www.color-blindness.com/coblis-color-blindness-simulator/) to assess how these two color codes would be perceived by protanopes and deuteranopes (Fig R1). The results suggest that our original color code can be easily distinguished by people affected by protanopia or deuteranopia (which are by far the most common forms of color-blindness). The color code using CUD colors does not seem to yield greater differentiability by these color-blind groups. Hence, we believe that our original color code is an acceptable choice.

It is worth noting that the most important distinction is between blue (bivariate) and red (multivariate), which are used to indicate these two different kinds of measures of synchrony or phase relationships. Green is used to indicate univariate measures of firing intensity and variability, which have been included to assess potential covariation of the measures of synchrony with these univariate measures. These measures tend to be grouped among themselves early in the hierarchical clustering procedure, and are also easily recognized as univariate measures by their names by most readers (e.g. firing rate, ISI CV, ISI local variation, ISI irregularity, etc.).

In the previous version of our manuscript, we included a legend for the MSTM type colorcode only in Fig 2, Fig 3, Fig 11, Fig S1 and Fig S3. In this revised version, we included it also for Fig 4, Fig 5, Fig 6, Fig 9, Fig 10, Fig S2, Fig S4, Fig S5, Fig S8, Fig S9, and Fig S10.

univariate	univariate
bivariate	bivariate
multivariate	multivariate
univariate	univariate
bivariate	bivariate
multivariate	multivariate
univariate	univariate
bivariate	bivariate
multivariate	multivariate

Figure R1: Color code comparison. The left column shows our original color code, and the right column shows a color code using colors suggested in the Color Universal Design (CUD) palette. The top row shows the normal (trichromatic) view, while the middle and bottom rows show simulations of how the color codes would appear to two frequently occurring categories of dichromats (protanopes and deuteranopes, respectively).

Reviewer 1 wrote:
"Fig 5 – drop either A or B, I'm not sure you need both."

Answer:
Fig 5 shows the effects of sample size variations in time (A) or space (B) for 5 illustrative MSTMs. One of the points conveyed by this figure is that, for many measures, the effects of sample size variation in space and time can be very different, as stated in subsection "Effects of time window length and number of neurons":

"The effects of sample size variations in space and time are drastically different, to the extent that some of the measures that display the smallest bias when subsampling in space are among those that exhibit the largest bias when subsampling in time, and vice versa (Fig 5 and 6, compare A with B)."

Reviewer 1 wrote:
"Fig 6 – I found this very dense and hard to follow; suggest looking at it again to think about the key point you want to make from this."

Answer:
We agree with the Reviewer that a lot of information is included in Fig 6, particularly in panels A and B. We designed this figure for conveying information at multiple levels of detail.

1. At the coarsest level, this figure can be read by focusing exclusively on the average values, indicated by the bars and used to rank MSTMs along the x axis.
2. At an intermediate level, readers can also take into account part of the information corresponding to individual spike trains, indicated with symbols. An important piece of information is whether a measure is consistently positively or negatively biased, or whether it can display both positive and negative biases, depending upon the synthetic spike train generation parameters.
3. At a finer level, readers can take into account symbol color, coding for synchrony level. This can inform on whether a given MSTM's bias or variability are systematically affected by the synchrony level (as in the case of variability for Kruskal correlation, the fourth measure from the right in Fig 6Bb), or whether they are display similar values regardless of the synchrony level (as it is the case for many MSTMs).
4. At an even finer level, readers can also take into account symbol type, which codes for spike generation formalism (single-scale or dual-scale), pseudo-rhythmicity, and sequentialness.

We acknowledge that the finest level of detail requires some level of magnification; however, the first three levels can be appraised directly at the normal magnification level, without effort. We believe that the richness of this figure is valuable, since it provides both an average-level view for the benefit of the general reader, as well as information at finer levels that enables specialized readers to focus on their specific points of interest (e.g., on one specific measure, or on the effect of synchrony strength on a subset of measures or across the whole MSTM library).

Reviewer 1 wrote:
"Fig 7 – the only thing I take from this is the skewing of the colour map by the yellow outliers in the bottom 6 rows, which makes it hard to make any insights from all the other rows. Do you also need to show all the columns (sites?)"

Answer:

We agree that the data presented in Fig 7 (as well as the corresponding data for the other two biological datasets, shown in Fig S6) exhibits some positive skewness that results in a suboptimal use of the color scale. In this revised version, we clipped values greater than $\max(-S_Z)$ to that value, and reported it in the caption as follows:

"In both (A) and (B), values greater than $\max(-S_Z)$, where S_Z indicates a generic MSTM value after z-scoring and the maximum value is taken over MSTMs and time windows, are clipped to that value for improved visualization."

Please note that this modification only affects the visualizations shown in Fig 7 and Fig S6. We believe that this modification results in a better use of the color scale, enabling a clearer appreciation of the extents of within- and across-recordings variability, and the types and prominence of dynamical features such as pseudo-periodicities and nonstationarities, as indicated in the text of this revised version:

"This representation provides a global depiction of a whole dataset, offering a direct impression of the extent of variability within- and across-recordings. It also illustrates the types and prominence of dynamical features of the data, including nonstationary and pseudo-periodic aspects, and the MSTMs that reveal them. For example, `site9` shows a nonstationary firing rate profile, which decreases slightly in the middle and increases markedly near the end of the session. A similar profile is observed for a number of other MSTMs, notably for vR and VP, which have been shown to depend markedly on firing rate. As another example, `site3` and `site7` display peaks and troughs across many MSTMs which tend to recur every 3–4 time windows."

We would prefer to retain all the recordings, since one of the points conveyed by this figure is to illustrate how the proposed method can be used for a global visualization of a whole dataset, enabling researchers to qualitatively infer several properties of the dataset (including variability within- and across-recordings, and types and prominence of dynamical features such as pseudo-periodicities and nonstationarities) at a glance.

Reviewer 1 wrote:

*"Fig 11 - the word clouds in D *might* be better represented as a table of figure directly reporting the relative frequencies; the word plots to me eye are just highlighting 3 measures.*

Stephen Eglen
[2024-10-18 Fri]"

Answer:

We thank the Reviewer for his suggestion. In this revised version, the information shown in Fig 11D is also presented in two separate tables, one for the MSTMs included in the top 1% most discriminative pairs (File S1), and another for the MSTMs included in the top 1% most synergistic pairs (File S2). We would prefer to retain Fig 11D since it might offer a more direct depiction of the same information, which some readers might find useful.

<u>Answer to Reviewer 2</u>

Reviewer 2 wrote:
"Reviewer #2: In this paper, the authors do analyse a battery of different measures for synchronization in both simulated and experimental neural data, and compare the differences, similarities, and advantages of each one of them. The paper is well-written and I believe that such a comparison can be useful for people working with neural oscillations. However, there are some issues that I believe should be tackled prior to the publication of the paper."

Answer:
We thank the Reviewer for the encouraging comments.

Reviewer 2 wrote:
"1) The generation of the synthetic spikes seems to cover a comprehensive set of possibilities for the analysis. Although one can think about more complex scenarios, I understand also the necessity to keep the trials simple. However, all synthetic neurons follow the same synchronous behaviour. I think it would be interesting to check what happens what happens when some neurons fire independently. I understand that this is partially captured by changing r0 and m, as having m « r0 would lead to small modulation over a background of spikes. However, all neurons participate in the modulation. I wonder if some of the synchrony measures could change their behaviour when the background firing rate is produced by neurons who do not engage in the global modulation. I also wonder if the authors have any insight about how correlations between neurons would affect some of the measurements, as the synthetic spikes are drawn independently, while structured functional connectivity might have an effect on some of the measurements, especially the ones that compute pairwise values, like Hunter-Milton similarity."

Answer:
We thank the Reviewer for the suggestion, and particularly for the interest in heterogeneous synthetic spike trains. In previous versions of the manuscript, we included additional synthetic spike train families that included neuronal heterogeneity parameterized similarly as suggested by the Reviewer. However, for the sake of brevity, we decided to remove that part from the current manuscript, and present it separately. Then, the manuscript was still deemed too long by the Editors, so we prepared a shortened version for review which was still deemed too long by the Editors and by Reviewer 1. Consistently with these advices, and considering the already extensive scope of the article, we still believe that the presentation of the heterogeneous synthetic spike train families and the behavior of the MSTM library on them could be better included in a separate article.

In our view, this article presents a minimal set of synthetic spike train families and biological datasets for illustrating our approach and the kind of insights that can be gained with a highly comparative approach to spike train analysis, with the intention of expanding the range of synthetic spike train families in the future, particularly with respect to heterogeneous spike trains and trains exhibiting spiking bursting activity.

Reviewer 2 wrote:
"2) Some of the measurements do aim to detect some special features, like structure in the spike trains. For example, setting r0 and modulation behaviour to asynchronous behaviour, the time-dependent SPIKE-distance is sensible to how the spikes are structured inside of the "bursts", altering SPIKE-distance. Hence, although this measurement might not seem the strongest detecting the modulation it might be useful for other purposes."

Answer:
We considered two different synthetic spike train formalisms to increase the generality of our results, decreasing the likelihood that our results are overly dependent on certain attributes of specific spike train formalisms. However, it is still possible that some of our results are partly dependent on these specific formalisms. To clarify this aspect, we added the following sentence in the Discussion, just after our consideration regarding the absence of an overall "best" measure:

"We considered two different synthetic spike train formalisms, simple enough to be described by just 5 parameters and adopting general assumptions based on simple and widely used statistical distributions, to increase the generality of our results. However, it is still possible that some of them could be partly dependent on these specific formalisms. In particular, it could be possible to design spike train formalisms that favor a particular MSTM, potentially yielding top performance for a MSTM that is not among those that correlate more closely and selectively with the parameters determining synchrony in our analyses."

Reviewer 2 wrote:
"3) The authors do cluster the data using hierarchical agglomerative clustering. From the text, I can deduce that they define a distance between nodes which is 1 - abs(correlation) and clustered using that as a distance. However, there is no explanation on which software/algorithm they use to construct the dendrogram from the distances, which should be explicitly stated. I also would like to know if the authors have considered other ways to cluster the measurements, such a nested stochastic block matrix community detection."

Answer:
We used hierarchical agglomerative clustering with average linkage clustering using MATLAB's `linkage` function. In this revised version, we included additional details in subsubsection "Hierarchical clustering of measures" in the Supplementary Methods:

"Then, an iterative procedure was conducted to group MSTMs into a hierarchical cluster tree or dendrogram, using MATLAB's `linkage` function. We started with each MSTM as its own cluster. Then, the two closest clusters were joined together into a single cluster, and this procedure repeated iteratively until all the MSTMs were grouped into one cluster. The distance between two clusters A and B was defined as the average among the distances between all pairs of MSTMs (S_i, S_j), where $S_i \subseteq A$ and $S_j \subseteq B$ (average linkage clustering)."

In early phases of this project, we did consider alternative linkage methods, in particular the single linkage clustering. Finally, we opted for presenting the results corresponding to average linkage because it is a widely used method, and because single linkage clustering can result in elongated clusters. In any case, the results obtained with single linkage clustering were not meaningfully different. We did not consider nested stochastic block matrix community detection. While it is possible that slightly different results could have been obtained with different methods, the close correspondence between the clustering results as depicted in the dendrograms of Fig 4A and Fig 8A and the corresponding block diagonal (or nearly block diagonal) structure of the similarity matrices shown in Fig Fig 4B and Fig 8B indicates that the clustering algorithm we employed is appropriate for the organization of MSTMs based on their empirical properties (i.e., based on the output they produce in the synthetic and biological spike trains considered).

Reviewer 2 wrote:
"4) I find figure 6 to be very complex. To start with, mean values of bias/variability are plotted with bars, and individual measurements are shown with dots of different shapes, depending on the type of timeseries

analysed. I do not understand how, e.g. ISI CV2 has dots both above and below zero and the mean clearly coincides with the values below zero. Am I missing something?

I also believe that they authors try to convey too much information. It seems that e.g. the bias is consistent over different types of timeseries, including single or dual scale, rhythmic and non rhythmic... so all this information could be just simplified and do a violin plot over all the cases, couldn't it? If I cannot read the figure or distinguish each single case among all the points then it should be pointless to add so much elements to the figure."

Answer:
We agree with Reviewer 2 that a lot of information is included in Fig 6, particularly in panels A and B. We designed this figure for conveying information at multiple levels of detail.

1. At the coarsest level, this figure can be read by focusing exclusively on the average values, indicated by the bars and used to rank MSTMs along the x axis.

2. At an intermediate level, readers can also take into account part of the information corresponding to individual spike trains, indicated with symbols. An important piece of information is whether a measure is consistently positively or negatively biased, or whether it can display both positive and negative biases, depending upon the synthetic spike train generation parameters.

3. At a finer level, readers can take into account symbol color, coding for synchrony level. This can inform on whether a given MSTM's bias or variability are systematically affected by the synchrony level (as in the case of variability for Kruskal correlation, the fourth measure from the right in Fig 6Bb), or whether they are display similar values regardless of the synchrony level (as it is the case for many MSTMs).

4. At an even finer level, readers can also take into account symbol type, which codes for spike generation formalism (single-scale or dual-scale), pseudo-rhythmicity, and sequentialness.

We acknowledge that the finest level of detail requires some level of magnification; however, the first three levels can be appraised directly at the normal magnification level, without effort. We believe that the richness of this figure is valuable, since it provides both an average-level view for the benefit of the general reader, as well as information at finer levels that enables specialized readers to focus on their specific points of interest (e.g., on one specific measure, or on the effect of synchrony strength on a subset of measures or across the whole MSTM library).

If we removed the symbols and only showed the bars, the information at the coarsest level would not be clearer than in the current version (where measures are ordered according to increasing bias (a) or variability (b), and background light/dark gray shading is used to indicate regions of good, intermediate, and poor behavior), but the information at levels 2-4 would be completely lost.

As the Reviewer correctly indicated, ISI CV2 can exhibit both positive and negative biases resulting from a finite time window length (panel Aa), and the mean value appears close to the bulk of negative values. An appreciation of the relative magnitude of individual values and their resulting mean value requires recognizing that we are using a symmetric log scale, since bias can be positive or negative and spans several orders of magnitude. In the particular case of ISI CV2, the mean value among the positive biases was 0.11, and the mean value among the negative biases was -0.21. Negative biases were more frequent than positive biases, resulting in an overall mean bias of -0.077, which appears relatively close to the mean value among negative biases when plotted with a symmetric log scale.

We added the following note in the caption to improve clarity:

"Note the use of a symmetric log scale for bias in (a) and (c)."

Reviewer 2 wrote:
"For Ac and Bc, as far as I understand the best would be to have no bias and no variability, so a point which is very low in the y axis while being in the center of the figure, close to bs=0. I would at least mark the bs=0 or visualize where an "ideal measure" would be in the variability-bias axis. Readers might think at first glance that bottom left corner is best."

Answer:
We thank the Reviewer for the useful observation. We added a vertical line indicating $\mathcal{B}_S = 0$ in Fig 6Ac and Bc, and in the corresponding panels of Figs S4 and S5, and added this line in the caption of Fig 6:

"Variability is plotted against bias in (c); best behavior corresponds to minimum $\mathcal{V}_S$ and $|\mathcal{B}_S|$."

Reviewer 2 wrote:
"5) For the analysis of the data, in particular Fig 7 and 8, if I understand correctly what the authors do is to compute the correlation matrix between each pair of timeseries, then get the distance matrix and cluster it, and then order the time windows according to the resulting clustering in fig 7. I do not understand what is the logic behind this analysis, as it is just a reordering of the time windows that has nothing to do with the measurements. Then, the text suggests that one applies the measurements to the time windows and then projects the high dimensional vectors to a 2D space (lines 574-577), referencing Fig 8C, but caption of Fig 8 clearly says that this representation is just obtained by the correlation between time windows, i.e. no synchrony measurement has been applied in there. The conclusion of the whole figure, also, is that time series of same session are more similar to each other than those between different sessions, which seems to be very intuitive. What do the authors intend to show here, and how does the battery of measurements enter into this analysis? From the way it is written it is very difficult to understand."

Answer:
We thank the Reviewer for indicating this potential source of confusion. All the results we present with respect to clustering over time windows used patterns resulting from the application of the core set or the extended set of MSTMs, either directly or after the application of MDS dimensionality reduction.

The details of the hierarchical clustering procedure over time windows are reported in the Supplementary Methods (subsection "Analyses of the relationships between measures and between time windows", subsubsection "Hierarchical clustering of time windows"):

"To organize and visualize the structure of neuronal activity and coordination patterns in biological spiking data, as captured by our MSTM library, we conducted hierarchical agglomerative clustering analyses on time windows, similarly as performed on MSTMs. Each time window was described by a feature vector resulting from the application of each MSTM. As a distance metric, we used $D = 1 - \rho$, where ρ is the Spearman correlation value between each pair of time windows. Linkage was performed using the average linkage criterion."

In this revised version, we remind readers of our approach just before presenting the clustering results over time windows (subsection "Analyzing experimental data through the lens of the highly comparative approach", subsubsection "Spike train variability and recording-wise distinctiveness"):

"To organize the structure of neuronal activity in biological spiking data, as captured by our MSTM library, we conducted hierarchical agglomerative clustering analyses on time windows, with each time window represented by a feature vector derived from the application of each MSTM."

Reviewer 2 wrote:
"6) For the discriminative analyses, although it seems true that pairing two observables can in average improve the classification power, it is not true that this happens for all observables, and especially it does not happen for the ones that perform best individually. The Tiesinga-Sejnowski synchronization already gets an accuracy larger than 0.98, according to the authors, which is basically the same to the best performing pair, which includes the TS distance. The authors do compute the synergy between pairs to see how much does the pairing improve each individual observable's results. It turns out that the most discriminative ones have a synergy close to 0 (Fig 10B), which means basically that doing the pairing does not make a big difference. Actually, many values in the synergy matrix are low."

Answer:
The Reviewer's observations are correct. We made similar observations in subsubsection "Brain state classification from spike train measures":

"The most synergistic pairs comprise MSTMs that do not perform particularly well at the individual level, but provide synergistic information when considered jointly (Fig 10 and Figs S9 and S10, compare top and bottom panels, and Fig 11C)."

"However, certain MSTM pairs exhibit negative synergies, indicating impaired MSTM pair decoding in comparison to the most performing of the individual MSTMs comprising the pair."

and in the Discussion:

"In the particular dataset we considered [93], decoding accuracy is high even when MSTMs are considered individually, and increases only slightly when considering MSTM pairs (from 0.982 to 0.989 in the best recording, and from 0.789 to 0.825 at the population level for within-recording decoding; and from 0.783 to 0.805 for across-recording decoding)."

Reviewer 2 wrote:
"7) Finally, I would like to tackle the issue with the code. The analysis presented by the authors really needs to ensure that all metric are correctly implemented, and that if there is any potential technical choice to implement the measurement this is clear for replicability.

First of all, the code is really messy. I wanted to check the implementation of some of the measurements I am familiar with. It seems that each observable has its own Matlab file, but those are mixed with other data analysis procedures inside the data_analysis folder, and some of the filenames do not coincide with the paper name (I could not find any "mean phase coherence", for example) and there is no explanation on how the measurements were implemented. It is mentioned that implementation is following the authors' guidelines in each paper, but in the accompanying code it would be useful to have a good explanation for each file."

Answer:
As stated in subsection "Multineuron spike train measures",

"All MSTMs have been implemented as described in the original publication, using the code provided by the authors when available, with the exception of the Tiesinga–Sejnowski synchrony measure S_{TS} [46]"

In those cases where the code provided by the authors of the original publication was used, it was not

modified, hence the original function name was also preserved. With respect to the code written by us, the measure name is included in the function name, albeit sometimes acronyms or other abbreviations were used. For example, the measure mentioned by the Reviewer is implemented in the function `phase_coherence_ei`.

To facilitate the retrieval of the functions corresponding to each individual MSTM, we added a comment with the exact same name as reported in the first column of Table 1 just before the block of code that computes it in `analyze_sinosc_train_ms.m`.

We created a directory named `MSTMs` inside `data_analysis` and moved there the functions written by us that calculate the MSTMs (https://github.com/GNB-UAM/sync_osc_MSTMs/tree/main/data_analysis/MSTMs).

Please note that the MSTM functions written by other authors are included in their respective folders under `third-party`, since they retain their respective licenses, and license validity is specified folder-wise (in this regard, please see also our reply to the licensing issue below).

We included a description for each high-level script and function in the README file (https://github.com/GNB-UAM/sync_osc_MSTMs/blob/main/README).

Reviewer 2 wrote:

"This would be useful first to double check the results, if needed, and for other people who wish to implement any of the measurements indicated here, or to compare between implementations, as there are usually some subtleties. For example, I wanted to check the mean phase coherence because this can be computed by performing a Hilbert transform or a linear interpolation between 0 and 2pi for each pair of spikes. The paper referenced by the authors uses the Hilbert transform. However, sometimes the second method can be more sensitive to synchronization, depending on the data's excitability."

Answer:

We used the second method, which is a more natural implementation for point processes. Reference [64] is the first publication that proposed Mean Phase Coherence (under that name). In that work, MPC was proposed as a measure of phase synchronization for continuous data, and hence required the application of the Hilbert transform for obtaining a phase signal. The point-process version of the measure is frequently used for the analysis of spike trains (e.g., as in Bogaard et al. 2009 J Neurosci) under the same name, albeit we note (in a footnote to Table 2) that the same measure was previously proposed in [86], and is also known as the Kuramoto order parameter.

Reviewer 2 wrote:

"According to the authors, the code could be use as a community library for synchronization measurements (969 - 978). I am afraid that in its current state, this is not possible, for several reasons.
- The code is not packaged correctly. It should be done as a MATLAB add-on, with files and dependencies perfectly organized and installation instructions."

Answer:

We believe that a GitHub repository is a more suitable way to share our code, as it better supports collaborative development. A MATLAB add-on could be a potential alternative. However, please note that some of the code used in this project is already available as a MATLAB add-on, as in the case of `export_fig`, and providing a MATLAB add-on that includes already existing MATLAB add-ons as subcomponents presents challenges in terms of distribution, maintenance, dependencies, licensing and version control. These challenges are simplified if GitHub is used as the platform for sharing our code. Furthermore, GitHub provides robust and effective tools for version control, including the possibility of

creating and managing forks. These features are not available for MATLAB add-ons.

Installation requirements and instructions are detailed in the README file (https://github.com/GNB-UAM/sync_osc_MSTMs/blob/main/README).

Reviewer 2 wrote:

"- *The code is lacking automated unit tests, that would be very useful for the authors to check if the measurements are computed correctly or not. Most of the measurements would yield a simple number in trivial cases, like completely random spikes or perfectly well synchronized bursts. These would help reviewers to check correctness in the code and will prevent the appearance of bugs if a user submit a pull requests that edits an existing measurement.*"

Answer:

Some measures do yield a simple number in the case of perfectly synchronized spike trains (as is the case for Mean Phase Coherence, which returns 1 for perfect synchrony), but not in the case of completely random spikes, because of finite sampling. We now provide a unit test for the example case of Mean Phase Coherence (https://github.com/GNB-UAM/sync_osc_MSTMs/blob/main/data_analysis/script_test.m), and encourage aspiring contributors to provide unit tests for newly proposed measures (see also our reply on the Contributing Guidelines below).

Pull requests that edit an existing measurement are discouraged. Bug reports on an existing measurement are encouraged. Alternative implementations of an existing measure are only considered if they yield a substantially different output in non-trivial cases (with empty spike trains or perfectly synchronized spike trains being considered as trivial cases), and should be included under a different name. This information is now included in the Contributing Guidelines, as detailed below.

Reviewer 2 wrote:

"- *Then, the 'third-parties' folder of the github repository includes a copy paste of all the code of external libraries used by the authors. This is problematic, as final users might have those libraries/code already installed and in different versions. Good packaging would ensure that the user knows all the dependencies and that those are installed following a pipeline adequate to the environment.*"

Answer:

We agree with the Reviewer's observation. In the revised version of the code, the python packages FOOOF and DADApy are no longer included directly, but installation instructions using `pip` are included in the README file. Third-party MATLAB packages such as `export_fig` are still included. If a user runs our `startup.m` file without modifications, the MATLAB packages included in the `third-party` directory will be used, but that can be customized by excluding specific packages with `genpath_exclude` (example usages are provided as comments in `startup.m`).

Reviewer 2 wrote:

"- *Another problem of copy-pasting is breaking the license. Most of the code that the authors include is GNU licensed allows personal use as long as license is shared. However, other includes no license, meaning it is 'all rights reserved'. Formally speaking, the authors are not entitled to reupload these files without explicit permission of the original authors. If the authors decide to keep the 'third party' folder library, they would need to change their LICENSE file to say that code inside of this folder is not covered by the license written here.*"

Answer:
We thank the Reviewer for indicating this issue. We shared our repository again, but now the GPL-3.0 license applies only to our original code, and the packages that are made available in the directory `third-party` retain their respective licenses, as indicated in https://github.com/GNB-UAM/sync_osc_MSTMs?tab=readme-ov-file#sync_osc_mstms.

Reviewer 2 wrote:
"- Finally, there is no code style for contributions, which should be required for any collaborative project."

Answer:
In this revised version of the code, we expanded the Contributing Guidelines section in README.md with more specific instructions (https://github.com/GNB-UAM/sync_osc_MSTMs/blob/main/README.md#contribute).

Reviewer 2 wrote:
"These issues prevented me from double checking several things I wanted to for this paper, and definitely users have no incentive to contribute to such a codebase rather than to reimplement the things themselves or use the original's paper implementation. Although not all my previous points would need to be implemented, I hope that the authors see why this codebase does not promote any community growth. It is also fine if the authors just want to share the code as-it-is for reviewing purposes, as not everything needs to be a nice package, but then they should not sell this in the paper."

Answer:
We thank the Reviewer for the constructive feedback on our code. We believe that the changes detailed above improved the usability of our code, yielding a codebase that can better contribute to community growth.

Reviewer 2 wrote:
"In general, I think the study can be interesting, but has some major issues, especially concerning the high-dimensionality analysis and the discriminative analysis, points 5 and 6. The comparison of different observables is fine, although figures can be improved (point 4) and the code is right now not usable by a community (point 7). All together, this weakens a lot the punchline that the authors are trying to deliver, and I cannot recommend this paper for Plos comp bio in its current form. I might reconsider after author's responses especially points 4-6."

Answer:
We thank the Reviewer for the useful feedback. We believe that our responses to the indicated points solved the concerns raised.

We renew our acknowledgments to the Referees for the useful comments and to the Editors.

Best regards,

Fabiano Baroni & Ben D. Fulcher.
