# Supplementary material for: Synchrony, oscillations, and phase relationships in collective neuronal activity: A highly comparative overview of methods
Source: PLoS Comput Biol. 2025 Oct 24;21(10):e1013597. doi: 10.1371/journal.pcbi.1013597 (PMC12574956; doi:10.1371/journal.pcbi.1013597)
Supplement: Attachment — Submitted filename: sync_osc_ms_letter.pdf [file pcbi.1013597.s018.pdf]

July 28th 2025

Dear Editors,

We are pleased to learn that many of the Reviewers' concerns were resolved with our revised submission. In the following, we address the remaining concerns, and indicate the corresponding changes that have been made to our manuscript.

Answer to the Editors' Comments

The Editors wrote:
"The authors must still do more to simplify and condense this manuscript - in particular, they should edit Figure 6 to make it more clear, and endeavour to make the simple take-home message(s) more prominent in the main text. It seems that there are also some issues with the online code base, which should be accessible and well documented prior to acceptance. These changes will make the manuscript easier to read, the results easier to digest, the code easier to use, and therefore make this work more likely to cited by researchers actively working in this field."

Answer:
In this revised version, the discussion on the top-performing synchrony MSTMs, previously included in the Supplementary Discussion in Text S1, is now presented in the main manuscript, in the Discussion. The Discussion section now includes subsection headings for improved readability.

To further simplify the manuscript, we moved the most technical details of the Methods subsection "Multineuron spike train measures" to a dedicated subsection in the Supplementary Methods, titled "Multineuron spike train measures: supplementary details" (Text S1). We also made several changes throughout the whole manuscript to improve clarity and precision, and to further emphasize our most important messages.

As explained in more detail below, it is difficult to simplify Fig 6 without compromising its goal. In this revised version, this figure is included as a Supplementary Figure. This decision was made to improve readability, as the main figure of this subsection (Fig 5) and the accompanying text provide sufficient information to convey the main points of this subsection. This decision also aims to yield a shorter manuscript, considering that part of the Supplementary Discussion of the previous version is now included in the main manuscript.

We also revised the GitHub repository, clarifying the Contribution Guidelines and making the installation instructions more directly accessible.

Answer to Reviewer 1 (Stephen Eglen)

Reviewer 1 wrote:
"I thank the authors for their revised submission, and their detailed response letter.

Whilst the authors have considered each of the points I addressed in my review, in most of the cases it appears that they have decided not to adjust their manuscript. In some cases, this appears warranted, e.g. not removing the correlation index - citing the useful Peters et al (2018) recommendations. However, in other cases, it appears little has changed in the manuscript. For example, I still find figure 6 overly confusing."

Answer:
We acknowledge that Fig 6 conveys a wealth of information. It is difficult to simplify this figure without curtailing its richness. Modifying this figure by focusing on a subset of measures and/or a subset of synthetic spike train formalisms would miss the point that this figure is designed to convey, which is precisely to provide a broad depiction at multiple levels of detail of finite-size effects in both time and space for all MSTMs and spike train formalisms considered, along with their mean values across spike train formalisms. In addition, it is worth acknowledging that the corresponding information for a subset of MSTMs and particular spike train formalisms is already provided in Fig 5 and Fig S3.

In this revised version, this figure is now included as a Supplementary Figure (Fig S4). We believe that this choice improves the readability of our manuscript, since the information provided in the text and in Fig 5 is sufficient to convey the main points of this subsection: that MSTMs vary widely in the bias and variability of their estimates due to temporal and spatial finite-size effects; that the effects of sample size variations in space and time are drastically different, to the extent that some of the measures that display the smallest bias when subsampling in space are among those that exhibit the largest bias when subsampling in time, and vice versa; and which are the MSTMs that display the best and worst behavior in these assessments.

Including Fig 6 as a Supplementary Figure also ameliorates the length issue, especially considering that, in this revised version, the discussion on the top-performing synchrony MSTMs, previously included in the Supplementary Discussion in Text S1, is now presented in the main manuscript, in the Discussion, as indicated below.

Reviewer 1 wrote:
"I'm also surprised that the authors decided to keep the red-green colormap; at this stage it might be an editorial decision, bearing in mind advice:

https://journals.plos.org/plosbiology/article?id=10.1371/journal.pbio.3001161&rev=1

https://www.nature.com/articles/s41467-020-19160-7

https://colorbrewer2.org"

Answer:
We appreciate Dr. Eglen's advice on this important point. In this revised version, we adopted the ColorBrewer 3-class Dark2 qualitative palette, which is labeled as colorblind safe in the third link provided by the Reviewer, and is composed of the following colors:

- Teal (RGB: [0.106, 0.620, 0.467])

- Purple (RGB: [0.459, 0.439, 0.702])
- Orange (RGB: [0.851, 0.373, 0.008])

As in our previous response to Reviewers, we used the color blindness simulator Coblis (https://www.color-blindness.com/coblis-color-blindness-simulator/) to assess how our original color scheme and this ColorBrewer scheme would be perceived by protanopes and deuteranopes (Fig R1). The results suggest that the colors of both our original palette and the ColorBrewer 3-class Dark2 palette can be distinguished similarly well by trichromats, with the ColorBrewer palette perhaps slightly improving on the CUD palette we considered in our previous revision, particularly with respect to the distinction between teal and purple.

The protanopia and deuteranopia simulations show that the ColorBrewer palette is likely to be distinguishable to a satisfying degree by these populations as well, especially with respect to the distinction between purple (bivariate) and orange (multivariate), which is the most critical distinction since univariate MSTMs can be easily recognized as univariate measures by their names by most readers. Beyond the results of these simulations, we believe that the adoption of the ColorBrewer 3-class Dark2 is an adequate choice for colorblind safety, since this colormap has been explicitly designed to accommodate colorblind readers.

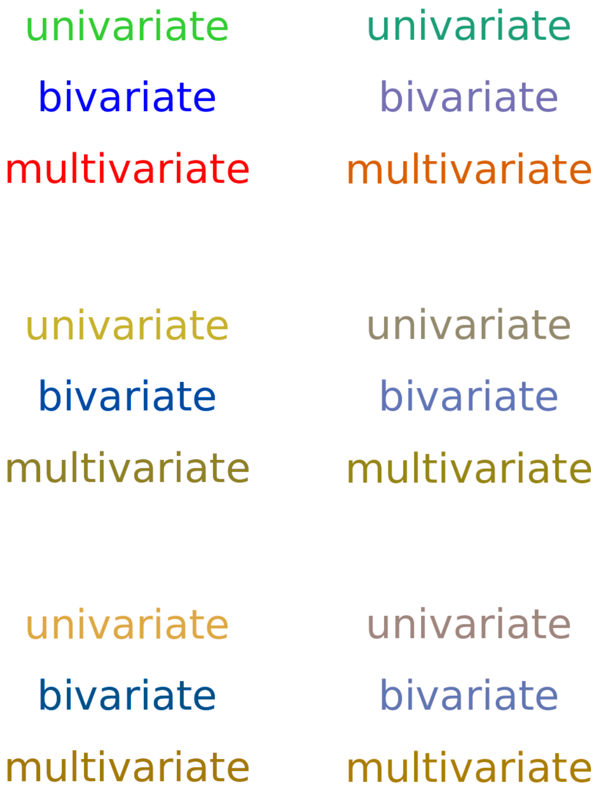

Figure R1: Color code comparison. The left column shows our original color code, and the right column shows a color code using colors suggested in the colorbrewer2 3-class Dark2 palette. The top row shows the normal (trichromatic) view, while the middle and bottom rows show simulations of how the color codes would appear to two frequently occurring categories of dichromats (protanopes and deuteranopes, respectively).

Reviewer 1 wrote:

“I note the editors suggested (quoted from page 1 of the response letter) that the "authors should endeavour to simply the manuscript"... and "draw clear conclusions from this survey". In its current form, I believe this has not been achieved. For example, the conclusions re: methods is relegated to supplementary text S1, rather than being in the main document. I feel that important text like is better placed in the main document.”

Answer:
We followed the Reviewer’s suggestion and now present the discussion on the top-performing synchrony MSTMs, previously included in the Supplementary Discussion in Text S1, in the main manuscript, in the Discussion. This change ensures that this key discussion is now featured in a prominent section of the main manuscript, enhancing its visibility and accessibility. The Discussion section now includes subsection headings for improved readability.

To further simplify the manuscript, we moved the most technical details of the Methods subsection "Multineuron spike train measures" to the Supplementary Methods (Text S1). We also made several changes throughout the whole manuscript to improve clarity and precision, and to further emphasize our most important messages.

Reviewer 1 wrote:
“My personal recommendation therefore is that the manuscript still needs to be significantly reworked to make it useful. To address the length issue, one suggestion might be to split the work into two parts – one describing the methods, and one describing the applications. However, that is also a lot of work.

Stephen Eglen
2025-04-21”

Answer:
We acknowledge the Reviewer’s suggestion, but we believe that our manuscript is better presented as a single manuscript with a single goal: compiling, benchmarking and organizing a comprehensive battery of MSTMs of synchrony, oscillations, and non-random phase relationships based on their behavior on both synthetic spike trains with known ground truth as well as biological spike trains. We acknowledge that our manuscript is longer than average, which reflects its comprehensive scope of providing a unified picture of multineuron spike train measures and their behavior on both synthetic and biological spike trains. To ameliorate the length issue, we have moved Fig 6 and the technical details of the Methods subsection “Multineuron spike train measures” to the Supplementary Material.

<u>Answer to Reviewer 2</u>

Reviewer 2 wrote:
"In general I am satisifed with author's responses to my comments. The authors have been quite detailed in their answers and I think the changes in the paper and especially in the code are notable. I have two minor comments that the authors may want to check prior to publication."

Answer:
We thank the Reviewer for the positive comment.

Reviewer 2 wrote:
"- First, about Fig 6. After the author's explanation it became a bit more clear. Still I believe that it conveys too much information, but since I was not able to find a satisfying solution myself after thinking about it for a bit, maybe this is the best way to do it. I think that the paper would benefit from including the answer given by the authors, even in a short way, help readers read the figure from the coarse to the fine details."

Answer:
We appreciate the Reviewer's suggestion. In this revised version, this figure is now included as a Supplementary Figure as Fig S4. We believe that this choice improves the readability of our manuscript, since the information provided in the text and in Fig 5 is sufficient to convey the main points of this subsection.

We now included the following sentence at the end of the caption of Fig S4:

"This figure conveys information on temporal and spatial finite-size effects at multiple levels of detail, from average values (bars) to individual results for each spike train formalism (symbols), with symbol color coding for the level of synchrony and symbol type coding for spike train formalism."

Reviewer 2 wrote:
"- Second, I understand the point of the authors regarding code organization and the negative to generate an add-on. As the authors point out, licensing and maintaining code is a complicated task. However, I do not agree with some of the decisions taken. One is that the authors seem to be very restrictive on what should be pull-requested to the repo, discouraging edits or even optimizations that are not 'substantial', which might hinder development. For instance, this is a problem with licensing and the third-party methods used by the authors. Licensing is a complicated issue and the paper would have benefitted from the full re-implementation of the methods, granting the authors full rights over the software and allowing also for a replicability test. I understand however that this is a lot of work and the community should help in this task, but the 'contribution guidelines' do not encourage this. I believe that the reimplementation of those methods should be actually encouraged."

Answer:
We appreciate the reviewer's concerns regarding licensing. Our project integrates code from multiple third-party sources, each of which is included with its original license. We believe this is the most appropriate approach for acknowledging the original authors' contributions while enabling open, collaborative development. Even if we reimplemented the code developed by other authors, it would necessarily follow the same logic, hence the original licenses would still be relevant. Including the original

code with its corresponding license ensures transparency, legal clarity, and proper credit. This is the same approach adopted in the `hctsa` project, which also includes packages from multiple sources, with each third-party package retaining its original license, and license validity specified folder-wise (https://github.com/benfulcher/hctsa/tree/main/Toolboxes).

Please note that the third-party code packages we include are associated to either a BSD, MIT, GNU GPL, or similar license, all of which are open source licenses that allow redistribution, modification, and derivative use, provided their respective conditions are met. These characteristics make them suitable for inclusion in a collaborative, extensible research codebase like ours.

Our contributions guidelines discourage pull requests that edit the core implementation of an existing measure, as well as pull requests that include modifications of an existing measure that do not yield different outputs or substantial improvements in computational efficiency. This policy aims to preserve the stability and reproducibility of the root repository, which are especially important factors for a codebase associated with a published article. That said, we fully support experimentation, reimplementation, and optimization efforts. We encourage users to explore such developments through independent forks, which allow maximum flexibility and innovation without compromising the stability of the reference implementation. To better reflect this intent, we have clarified the Contribution Guidelines (https://github.com/GNB-UAM/sync_osc_MSTMs#contribute), which now read:

"Pull requests that edit an existing measure are discouraged. Bug reports on an existing measure are encouraged. Modifications or alternative implementations of an existing measure are only considered as pull requests if they yield a substantially different output in non-trivial cases (with empty spike trains or perfectly synchronized spike trains being considered as trivial cases), or a substantial improvement in computational time, and should be included under a different name. All other modifications are encouraged as independent forks."

Reviewer 2 wrote:
"I also see that installation instructions are not present, even when they are mentioned in the README. The authors might want to add them."

Answer:
Requirements and installation instructions are provided in the README file (https://github.com/GNB-UAM/sync_osc_MSTMs/blob/main/README), as mentioned in README.md (https://github.com/GNB-UAM/sync_osc_MSTMs/blob/main/README.md):

"The README file includes the installation requirements and instructions, and a description of all high-level scripts and functions."

We now added a hyperlink to the README file to facilitate its retrieval.

Reviewer 2 wrote:
"Let me add that, contrary to the previous version of the manuscript, the code now looks good enough to start a collaborative effort. In my opinion it will need more work but I see the authors manage also CompEngine, which looks like a well-maintained repository, so they definitely have experience and know what needs to be done from this point on.

The paper looks technically correct and I think it should be accepted. I would like to thank the authors for their patience in gathering all the methods and writing the comparison, which I am sure will be useful to researchers studying neural synchronization. I also apologise for taking longer in returning a review of the manuscript both times, but I really wanted to read all the text and take a look at the code."

Answer:
We would like to thank the Reviewer for the constructive criticism that helped to improve our manuscript and codebase. We believe that, thanks to the Reviewer's feedback, we were able to lay the groundwork for a collaborative project for the systematic benchmarking and organization of spike train measures.

We renew our acknowledgments to the Referees for the useful comments and to the Editors.

Best regards,

Fabiano Baroni & Ben D. Fulcher.
